# Development of Novel pH-Sensitive Eudragit Coated Beads Containing Curcumin-Mesalamine Combination for Colon-Specific Drug Delivery

**DOI:** 10.3390/gels9040264

**Published:** 2023-03-23

**Authors:** Eman J. Heikal, Rashad M. Kaoud, Shadeed Gad, Hatem I. Mokhtar, Abdullah Alattar, Reem Alshaman, Sawsan A. Zaitone, Yasser M. Moustafa, Taha M. Hammady

**Affiliations:** 1 Department of Pharmaceutics and Industrial Pharmacy, Faculty of Pharmacy, Suez Canal University, Ismailia 41522, Egypt; 2 Faculty of Pharmacy, The University of Mashreq, Baghdad 11001, Iraq; 3 Pharmacy Department, Ashur University College, Baghdad 10047, Iraq; 4 Department of Pharmaceutical Chemistry, Faculty of Pharmacy, Sinai University-Kantara Branch, Ismailia 41636, Egypt; 5 Department of Pharmacology & Toxicology, Faculty of Pharmacy, University of Tabuk, Tabuk 71491, Saudi Arabiasawsan_zaytoon@pharm.suez.edu.eg (S.A.Z.); 6 Department of Pharmacology and Toxicology, Faculty of Pharmacy, Suez Canal University, Ismailia 41522, Egypt; 7 Department of Pharmacology and Toxicology, Faculty of Pharmacy, Badr University in Cairo, Cairo 11829, Egypt

**Keywords:** curcumin, colon-specific delivery, hydrogel beads, mesalamine, rat, ulcerative colitis

## Abstract

This research aims to develop a drug delivery system that effectively treats colitis while administering curcumin/mesalamine by coating alginate/chitosan beads with Eudragit® S-100 to target the colon. Beads were tested to determine their physicochemical characteristics. Coating with Eudragit® S-100 prevents drug release at a pH of less than 7; this was demonstrated by in-vitro release conducted in a medium with gradually varying pH to mimic circumstances in various regions of the gastrointestinal tract. This study examined the efficacy of the coated beads in treating acetic acid-induced colitis in rats. Results showed that spherical beads were formed with an average diameter of 1.6–2.8 mm, and the obtained swelling ranged from 409.80% to 890.19%. The calculated entrapment efficiency ranged from 87.49% to 97.89%. The optimized formula F13 (which was composed of mesalamine-curcumin active ingredients, Sodium alginate as a gelling agent, chitosan as a controlled release agent, CaCl2 as a crosslinking agent, and Eudragit S-100 as a pH-sensitive coating agent) demonstrated the best entrapment efficiency (97.89% ± 1.66), swelling (890.19% ± 60.1), and bead size (2.7 ± 0.62 mm). In formulation #13, which was coated with Eudragit S 100, curcumin (6.01 ± 0.04%) and mesalamine (8.64 ± 0.7%), were released after 2 h at pH 1.2; 6.36 ± 0.11% and 10.45 ± 1.52% of curcumin and mesalamine, respectively, were then released after 4 h and at pH 6.8. Meanwhile, at pH 7.4, after 24 h, approximately 85.34 ± 2.3% (curcumin) and 91.5 ± 1.2% (mesalamine) were released. Formula #13 significantly reduced the colitis, and this suggests that the developed hydrogel beads can be used for delivering curcumin-mesalamine combinations to treat ulcerative colitis after adequate research.

## 1. Introduction

The ideal oral colonic delivery method would expedite drug absorption in the upper gastrointestinal tract (GIT) [1,2] the stomach, and the small intestine, while drug release would be maximized within the colon or large intestine. Local treatment in the colon provides benefits, including the delivery of the medicine close to the target site and a reduction in systemic side effects [3,4].

The process of delivering medications from the stomach to the colon includes using pH-dependent polymers, timed-release dose forms, or carriers that enzymes can digest in the colonic microbiota. Based on pH measurements of the GIT, the idea of utilizing pH as a triggering factor for drug release in the colon was conceived. The pH in the small intestine (pH 5.5–6.8) is alkaline, while in the transverse colon, it rises to over pH 7 [2]. For example, Eudragit^®^ S-100 (soluble at pH > 7.0) is a popular coating polymer that enables the control of drug release in enteric dosage forms as its solubility changes depending on pH [5].

Mesalamine (MSZ) is often used as the first line of defense against ulcerative colitis and Crohn’s disease. MSZ has an anti-inflammatory effect, is the first choice for the treatment of inflammatory bowel diseases, and is used to treat mild to moderate cases because it is generally well-tolerated and safe [4]. It is a member of class IV of the Biopharmaceutics Classification System (BCS); it has low aqueous solubility, low permeability, and, hence, low bioavailability. Additionally, it undergoes first-pass metabolism in the liver, where 20–30% metabolizes away from the drug-rich colon. As such, it usually requiring high doses to achieve a therapeutic effect, which can cause serious side effects such as nephrotic syndrome and hepatitis; all these factors weaken its therapeutic effect. The mucosa of the upper GIT allows MSZ to be absorbed by the stomach and small intestine [6,7].

The polyphenolic compound curcumin (CUR) is extracted from turmeric *Curcuma longa* Linn and has been reported to treat inflammation, oxidative stress, and cancer. CUR has demonstrated antiproliferative action with various cancer cell types in vitro, including those from colon, liver, and prostate cancers. The primary mechanism for its anticancer effects is the ability of the curcumin to push cancer cells, including colon cancer, to undergo apoptosis without harming healthy ones One of the major issues with the material is that only 30–40% of the content of curcumin is soluble in water and different techniques must be applied to enhance its solubility [1].

Hydrogels, such as sodium alginate (SALG), have been widely employed in biomedical applications, particularly in drug delivery. Hydrogel beads have been used as drug carriers in colon-targeting and pH-sensitive coating polymers. By varying their network cross-link density, hydrogels can hold many drugs and control their gradual release [8].

Chitosan (CS), which is the deacetylated derivative of chitin with cationic charge, is another polymer used for this purpose. It is a biocompatible, antibacterial, and biodegradable polymer that has also showed a strong biological adhesion and has contributed to developing enteric delivery systems [9]. The hydrogel beads are expected to provide a positive impact on drug delivery because they do not only delay the gastric release of medications, but also remain in the colon for an extended period. It has been suggested that crosslinked CS/SALG hydrogel beads would effectively regulate the release of a MSZ-CUR combination [10].

Anionic copolymers of methacrylic acid and methyl methacrylate, such as Eudragit^®^ S-100, are very pH-dependent. Because of its unique pH-solubility profile, Eudragit^®^ S-100has been proposed for use in microencapsulation for controlled-release purposes. This polyacrylic resin is appropriate for drug targeting in the colon since it is pH-sensitive and soluble at pH 7 or above due to the availability of carboxylic acid groups. Hydrogel bead drug delivery systems used for the site-specific transport of MSZ and CUR have been developed using Eudragit^®^ S-100, a pH-responsive enteric polymer, for the efficient prevention of ulcerative colitis [11].

Developing pH-sensitive Eudragit^®^ S-100-coated CS-SALG (MSZ-CUR) beads for colon targeting was the goal of this work. First, we prepared a complex between the anti-inflammatory drugs (CUR and MSZ) and hydroxypropyl-β-cyclodextrin (HP-β-CD) for improving the low-content drugs’ water solubility, bioavailability, stability, and therapeutic efficacy. Second, we formulated this complex in sodium alginate-based hydrogel formula, in which chitosan was used as a release-controlling agent. A Eudragit^®^ S-100 coating was used on the beads to prevent medication loss in the upper GIT and to deliver drugs to the colon with expected synergistic action for the combination of MSZ-CUR against experimental ulcerative colitis in rats. The beads’ physical characteristics and the in vitro release of the drugs were assessed to test the best formula and then test their anti-inflammatory effectiveness against the rat ulcerative colitis model.

## 2. Results and Discussion

### 2.1. Phase-Solubility Analysis

**Figure 1:**
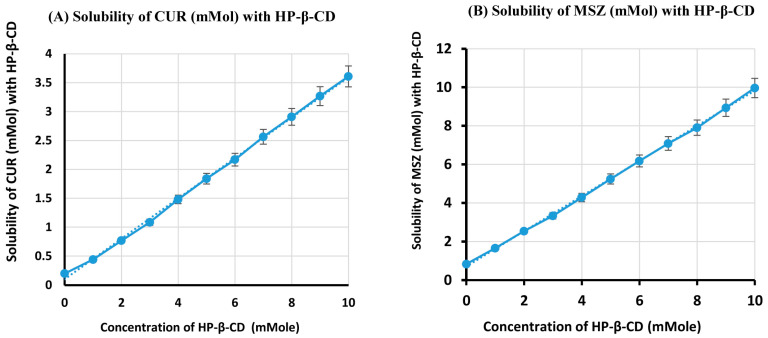
Phase-solubility diagram for the complex ingredients. (**A**) CUR with HP-β-CD, (**B**) MSZ with HP-β-CD systems using water at 37 °C.

Table 1 and Figure 1 show the stoichiometry of the drug/HP-β-CD inclusion complexes calculated using a phase-solubility curve analysis performed at 37 °C. The water solubility of CUR and MSZ increased linearly when the concentration of HP-β-CD increase over the studied range of concentrations, as indicated in the graphs. This linearity was distinguishing for AL-type and indicated that the inclusion complexes (MSZ/HP-β-CD and CUR/HP-β-CD) had binding stoichiometries equaling 1:1 [12]. Further, in the presence of 10 mM HP-β-CD, 11.96-fold and 18.41-fold water solubilities were observed for MSZ and CUR, respectively.

### 2.2. Characterization of MSZ/HP-β-CD Inclusion Complexes

Inclusion complexes between HP-β-CD and the drugs were indicated by comparison of the FTIR spectrums of the inclusion complexes with their corresponding spectra, acquired from their individual ingredients and physical mixture. Changes in the guest molecule’s distinctive bands, such as a removal of or reduction in peak strength, a wave number move, or the emergence of additional bands, may suggest complex formation [13].

**Figure 2:**
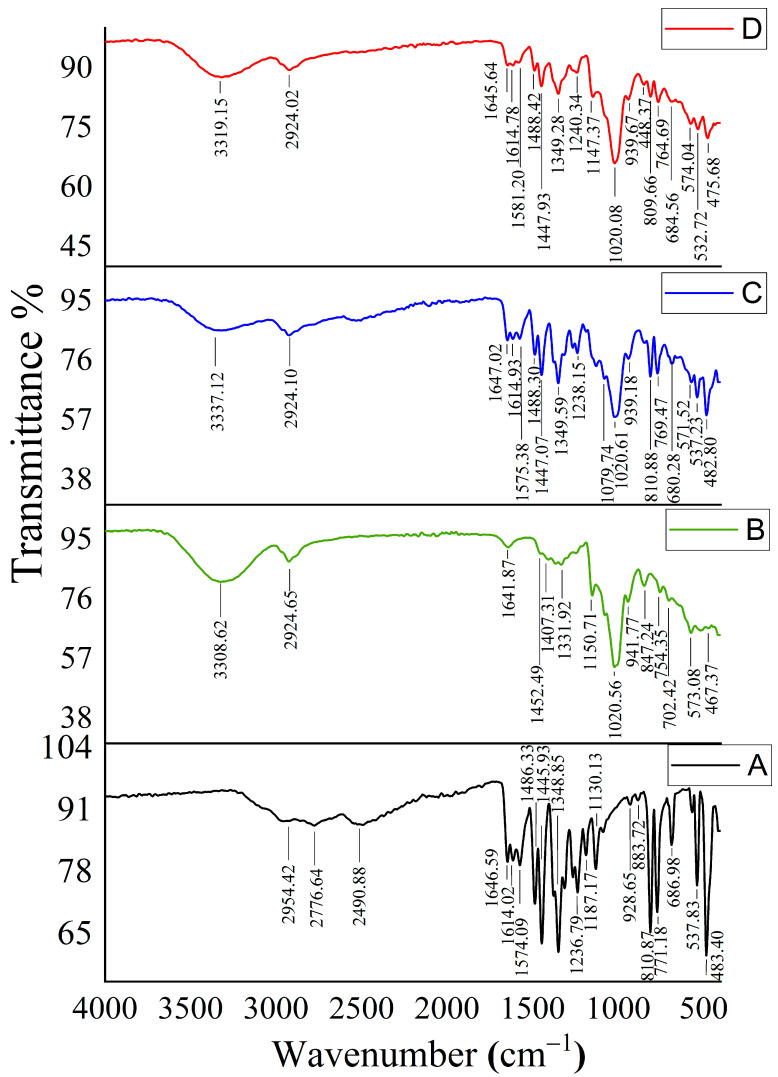
FT-IR spectra of each component of the inclusion complexes. (**A**) Free MSZ, (**B**) HP-β-CD, (**C**) HP-β-CD-MSZ physical mixture, and (**D**) MSZ/HP-β-CD inclusion complex.

Figure 2 shows the FTIR spectra of MSZ, HP-β-CD, the MSZ and HP-β-CD physical mixture, and the MSZ/HP-β-CD inclusion complex (Figure 2A–D, respectively). The obtained FTIR spectrum of MSZ (Figure 2A) demonstrated characteristic broad bands at 2954, 2776, and 2491 cm^−1^; sharp bands grouped at 1647, 1614, and 1574 cm^−1^; and another group of sharp bands at 1487 and 1446 cm^−1^. The acquired spectrum of HP-β-CD (Figure 2B) showed absorbance bands characteristic of an HP-β-CD FTIR spectrum, distinguishable from those of MSZ. The FTIR spectrum of the physical mixture of MSZ and HP-β-CD (Figure 2C) displayed the characteristic peaks for both MSZ and HP-β-CD in an additive pattern, with an expected decrease in the MSZ-characteristic bands’ intensity due to dilution with HP-β-CD. The FTIR spectrum of the HP-β-CD/MSZ inclusion complex (Figure 2D) was compared to the corresponding spectrum of the physical mixture of the two ingredients (Figure 2C). The comparison revealed a significant decrease in the presence of MSZ absorbance bands in the inclusion complex spectrum relative to that of the physical mixture for bands at 1645, 1614, 1488, and 1447 cm^−1^. In addition, there was a significant shift in the position of one band from 1575 cm^−1^ in the physical mixture to 1581 cm^−1^ in the inclusion complex, with a decreased extent of absorbance. These changes in the MSZ-characteristic bands indicated the insertion of MSZ into the HP-β-CD cavity to form the HP-β-CD/MSZ inclusion complex.

**Figure 3:**
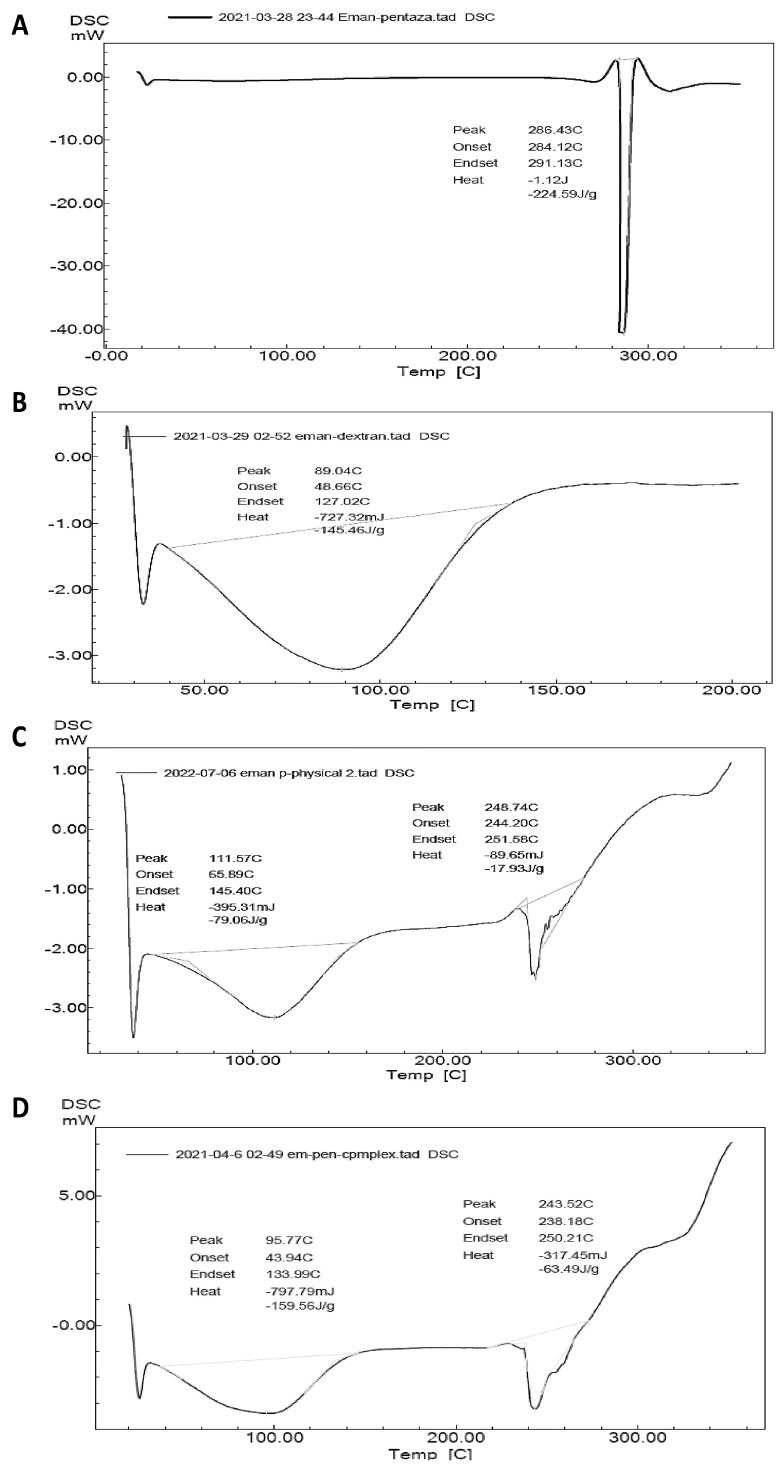
DSC thermograms of the components of the inclusion mixtures. (**A**) Free MSZ, (**B**) HP-β-CD, (**C**) MSZ and HP-β-CD physical mixture, and (**D**) MSZ/HP-β-CD inclusion complex.

For further confirmation of inclusion complex formation, DSC thermograms of MSZ, HP-β-CD, the MSZ and HP-β-CD physical mixture, and the HP-β-CD/MSZ inclusion complex were acquired (Figure 3A–D). Changes in the endothermic peak locations and intensities in either the physical mixture or the inclusion complex from those of the individual ingredients indicate a possible interaction between the studied ingredients. The DSC thermogram of MSZ presented a single endothermic peak at 286.43 °C (Figure 2A), corresponding to its melting point. On the other hand, the HP-β-CD thermogram demonstrated a single broad peak at 89.04 °C (Figure 2B). The thermogram of the MSZ and HP-β-CD physical mixture (Figure 2C) showed that the MSZ endothermic peak had shifted from 286.43 °C in pure MSZ to 257.23 °C in the physical mixture thermogram, indicating a possible interaction between the two ingredients. The thermogram of the HP-β-CD/MSZ inclusion complex showed a further shift in the MSZ endothermic peak location to 243.52 °C (Figure 2D). This observed shift supported the previous phase solubility and FTIR spectroscopy findings to indicate the formation of an HP-β-CD/MSZ inclusion complex.

### 2.3. Characterization of CUR/HP-β-CD Inclusion Complexes

**Figure 4:**
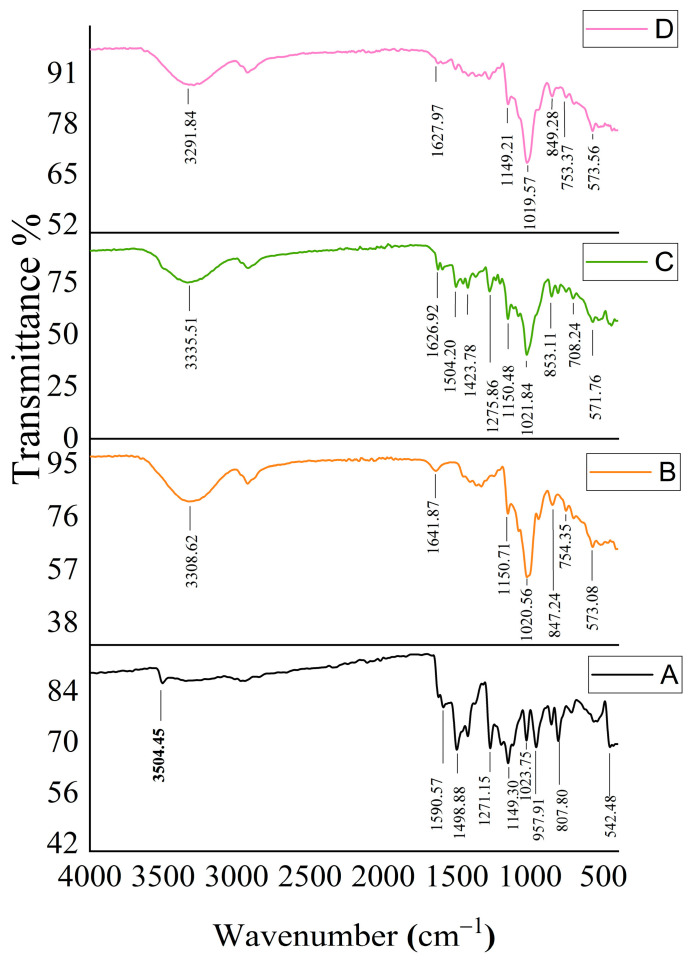
FT-IR spectra of the components of the inclusion complexes. (**A**) Free curcumin, (**B**) HP-β-CD, (**C**) curcumin and HP-β-CD- physical mixture, and (**D**) curcumin-HP-β-CD inclusion complex.

The FTIR spectra of CUR, HP-β-CD, the CUR and HP-β-CD physical mixture, and the CUR/HP-β-CD inclusion complex are provided in Figure 4. The FTIR spectrum of pure CUR ingredient (Figure 4A) displayed a weak band at 3504 cm^−1^, and strong bands of absorption at 1623, 1590, 1499, and 1424 cm^−1^. The obtained spectrum of CUR was distinguishable from that of HP-β-CD (Figure 4B). The acquired FTIR spectrum of the CUR and HP-β-CD physical mixture (Figure 4C) exhibited the characteristic bands of both CUR and HP-β-CD, with decreased intensity in the bands of both compounds due to their dilution. Upon comparison between the FTIR spectrum of the CUR/HP-β-CD inclusion complex (Figure 4D) with that of the physical mixture, the spectrum of the CUR/HP-β-CD inclusion complex showed the complete disappearance of the absorption band at 3504 cm^−1^ and a significant reduction in band intensity at 1504, 1455, and 1423 cm^−1^ relative to their corresponding bands in the physical mixture. These findings could be explained by the formation of an inclusion complex between CUR and HP-β-CD. 

**Figure 5:**
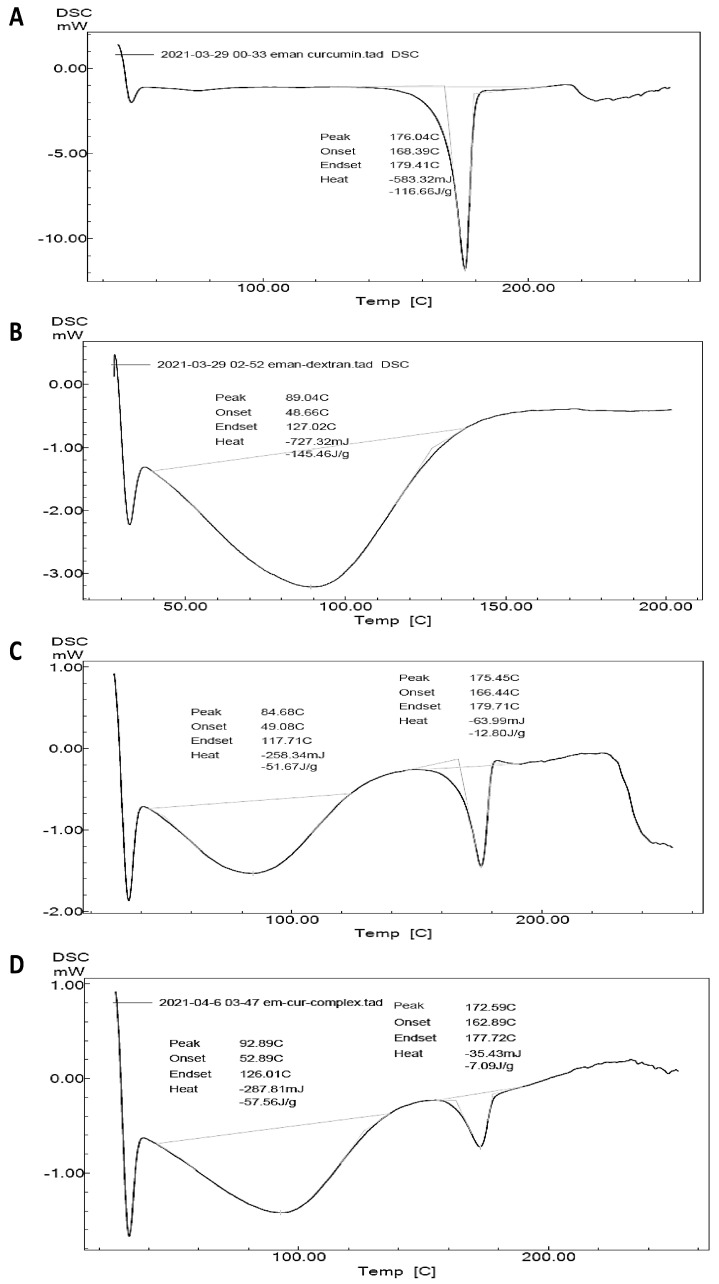
DSC thermograms of components of the inclusion complexes. (**A**) Free curcumin, (**B**) HP-β-CD, (**C**) curcumin and HP-β-CD physical mixture, and (**D**) CUR-HP-β-CD inclusion complex.

Further investigation into the possibility of inclusion complex formation between CUR and HP-β-CD could be inferred from the DSC thermograms of CUR, HP-β-CD, the CUR and HP-β-CD physical mixture, and the CUR/HP-β-CD inclusion complex (Figure 5A–D). The CUR thermogram demonstrated a single endothermic peak of high intensity at 176.04 °C (Figure 5A). On the other hand, HP-β-CD presented an endothermic broad peak at 89.04 °C (Figure 5B), distinguishable from that of CUR. The thermogram of the CUR and HP-β-CD physical mixture demonstrated a slight shift in the position of the CUR endothermic peak from 176.04 to 175.45 °C (Figure 5C), along with a significant reduction in peak intensity. The position of the CUR endothermic peak in the DSC thermogram of the CUR/HP-β-CD inclusion complex (Figure 5D) had changed significantly, moving to 172.59 °C, and had a lower intensity than the corresponding peak in the physical mixture. These findings indicated a possible interaction between CUR and HP-β-CD and supported the phase-solubility experiments and FTIR spectral analysis findings for the confirmation of inclusion complex formation.

### 2.4. Formulation Optimization Using D-Optimal Design

The factorial design has many uses and is highly flexible, which is important when comprehending drug development’s complexity. A variety of independent variables define the quantity of trials that must be run. Further, multiple regression analysis was here used to ascertain the results of each experiment. This study has a simple design with three variables and three experimental levels. We prepared the formulated batches in accordance with the plan and assessed for their various responses. The impact of the variable alone and combined with each response was investigated via multilinear regression analysis and ANOVA testing. All of the generated formulations (22 runs), with triplicate center points and statistical validation quadratic equation of the response’s diameter, swelling studies, and entrapment efficiency (Y_1_, Y_2_, and Y_3_, respectively), were constructed with the help of ANOVA using Design-Expert^®^ software (version 11). The response surface models (RSM), and their results were evaluated and validated via statistically significant coefficients with respective R^2^ values over the whole experimental region to find the compositions of the optimized formulation. Furthermore, the selected experimental domain and RSM equations were validated through the preparation of optimum checkpoint formulations and evaluated to find the optimized formulations.

#### 2.4.1. Bead Diameter

Table 2 shows that the hydrogel beads had an average diameter of 1.6–2.8 mm when measured with a vernier caliper.

#### 2.4.2. Swelling (%)

At pH 7.4, as shown in Table 2 below, the hydrogel beads swelled, leading to the bursting of beads containing even a trace quantity of alginate after only 2 h of testing. With Na^+^ in the buffer and Ca^2+^ in the Ca-alginate bead cavity, ion exchange can occur [14]. The same unexpected effect was found when examining the swelling of alginate beads at varying concentrations. The swelling behavior noticed in the dry beads was mostly linked to the hydration of the CS and SALG hydrophilic groups [15].

#### 2.4.3. Drug Entrapment Efficiency (%)

Determination of drug concentrations of the formulated peaks was performed by measuring the UV-vis absorbances at 306 nm and 421 nm, corresponding to MSZ and CUR, respectively. The measurement method’s selectivity regarding the beads’ matrices was confirmed as a measurement of the plain beads’ spectrum (Supplementary information, Figure S3A,B). The measurement method was found to be linear in the range of 4–12 µg·mL^−1^, corresponding to 40–120% of the measurement’s working concentration spectrum (Supplementary information, Figure S4A,B). Table 2 displays the computed values of entrapment efficiency and shows increased entrapment efficiencies. The higher loading efficiency of CS-SALG beads may be linked to their highly porous structure. Since the Ca^2+^ and -NH_3_^+^ present in CS compete, they can both react with –COO– in the structure of an alginate.

The bead diameters ranged from 1.6 to 2.8 mm, and the obtained swelling ranged from 410% to 891%. The range of entrapment efficiency extended from 87.5% to 97.9%. The effects of various factors are discussed separately. The observed responses of complex drug-loaded formulations can be found in Table 2.

**Table 2:** Different runs with changeable levels of independent variables and the resultant dependent variables (diameter, swelling and entrapment efficiency) of formulations.

Run	SALG (%)	CaCl_2_ (%)	CS (%)	Diameter (mm)	Swelling (%)	Entrapment (%)
F1	1	4	0.5	1.6 ± 0.20 *	455.55 ± 63.83 *	93.03 ± 2.02
F2	3	2	0.5	2.5 ± 0.17	800 ± 40.4	92.37 ± 0.26
F3	2	3	0.3	2.3 ± 0.44	507.47 ± 59.95 *	92.35 ± 0.02
F4	1	2	0.5	1.6 ± 0.26 *	488.23 ± 7.12 *	87.49 ± 1.17
F5	3	3	0.1	2.6 ± 0.30	738.09 ± 34.34	93.35 ± 1.65
F6	3	4	0.1	2.8 ± 0.26	743.13 ± 54.05	95.23 ± 1.10
F7	1	3	0.1	1.7 ± 0.17 *	470 ± 62.5 *	89.57 ± 1.94
F8	2	3	0.3	2.3 ± 0.20	529.63 ± 54.68 *	91.85 ± 1.60
F9	1	4	0.1	1.9 ± 0.20	472.72 ± 45.16 *	94.36 ± 2.00
F10	2	2	0.1	2.6 ± 0.36	500 ± 19.77 *	92.43 ± 1.12
F11	1	2	0.3	1.9 ± 0.44	409.80 ± 9.49 *	88.49 ± 1.08
F12	2	3	0.3	2.4 ± 0.26	507.84 ± 27.82 *	92.39 ± 0.64
F13	3	4	0.5	2.7 ± 0.26	890.19 ± 16.99	97.89 ± 1.61
F14	3	4	0.5	2.7 ± 0.50	900 ± 26.97	97.68 ± 0.38
F15	2	4	0.1	2.4 ± 0.46	517.64 ± 5.06 *	97.64 ± 1.61
F16	2	2	0.1	2.6 ± 0.17	505.76 ± 30.93 *	91.88 ± 0.89
F17	3	2	0.1	2.7 ± 0.56	757.14 ± 20.67	96.05 ± 0.66
F18	1	4	0.5	1.6 ± 0.17 *	457.69 ± 20.5 *	93.28 ± 1.01
F19	1	2	0.5	1.6 ± 0.17 *	490 ± 19.8 *	87.50 ± 0.65
F20	3	2	0.5	2.5 ± 0.36	792.15 ± 29.31	92.68 ± 1.61
F21	3	2	0.3	2.8 ± 0.61	834.57 ± 30.52	93.11 ± 0.4
F22	1	4	0.3	1.6 ± 0.30 *	423.81 ± 30.98 *	92.15 ± 0.34

Data are mean ± SD, *n* = 3. *: versus Formula #13 at *p* < 0.05.

### 2.5. Developing Optimization Models

A criterion based on the attractiveness of a formulation was used to determine the optimal response values. All of the results came from the program’s built-in numerical analysis tool. The model’s accuracy was ensured by creating a formulation of the complex pharmaceuticals put into beads in accordance with several variables posited via an overlay plot. Multiple responses, including entrapment efficiency (%), bead size (mm), and swelling (%), were measured to determine the optimum formulation (F13). We compared the detected responses with the predicted responses and estimated the error percentage, as shown in Table 2. The optimal formulation of complex drugs (F#13) showed an entrapment efficiency of 97.89 ± 1.66%, swelling of 890.2 ± 60%, and a bead size of 2.7 ± 0.8 mm. A lower % of errors shows that the produced model was the best fit model for D-optimal designs.

#### 2.5.1. Model Fit Report for the Effect of Sodium Alginate

**Figure 6:**
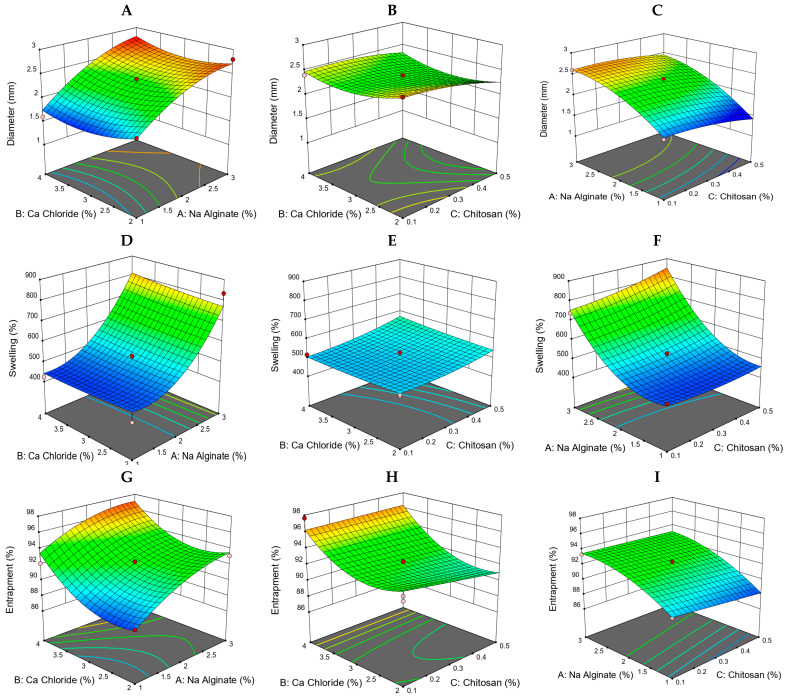
Response surface graphs for the impact of variables. (**A**–**C**) Response surface graphs representing the impact of various variables on the diameter of the formulations. (**D**–**F**) Response surface graphs representing the impact of various variables on swelling. (**G**–**I**) Response surface graphs representing the impact of various variables on the bead % entrapment efficiency.

As the concentration of SALG rose, the diameter also increased (Figure 6A,B), which was consistent with results obtained previously [6]. Model graphs revealed that SALG dramatically affects formulations’ swelling (Figure 6D,E). As the concentration of SALG rose, the swelling of the formulations also rose, as presented previously in [16]. where a trend of increasing diameter with a corresponding increase in swelling was observed. The model graphs demonstrated that SALG also profoundly affects the formulations’ encapsulation efficiency. Figure 6G,H displays that, as the SALG concentration was elevated, the formulation’s encapsulation efficiency was also elevated. This seems to be consistent with findings obtained previously [17]. All of the formulations benefited greatly from a higher SALG concentration regarding encapsulation efficiency.

#### 2.5.2. The Effect of Calcium Chloride

The model graphs in Figure 6A,C indicate that the diameter of the response surface decreases as the calcium chloride concentration rises. This can also be observed in the model graphs (Figure 6A,C). The increase in calcium chloride concentration led to greater swelling of the formulations (Figure 6D,F). At pH 7.4, we observed that the maximum swelling ratios rose with the concentration of CaCl_2_, and disintegration took place earlier for those beads formulated in low CaCl_2_ solution compared to other concentrations of CaCl_2_ solution. During bead preparation, the concentration of Ca^2+^ may influence the density of beads’ cross-linking. This result agrees with the findings obtained by another research group [18]. It is evident that the entrapment efficacy increased when the CaCl_2_ concentration was increased (Figure 6H,I) because the cross-linking reaction was favored [19].

#### 2.5.3. The Impact of Chitosan on Diameter and Entrapment Efficacy

The increasing concentration of CS resulted in an increment in the diameter of formulations [20]. This rise in formulation diameter may be attributable to a concomitant increase in the mean diameters of the beads, implying the production of a thicker CS layer, as seen in Figure 6C. The increased CS concentration leads to increased swelling of formulations (Figure 6F). The swelling index was significantly amplified with an increased CS concentration at pH 7.4, but in basic conditions, CS-SALG beads swelled the most due to the presence of ionized carboxylic groups, which are highly hydrophilic. As a result, it is possible to conclude that CS-SALG beads could shield the medicine from the extremely acidic environment of the stomach. This outcome is consistent with findings obtained previously [21]. Because of the impact of CS concentration on the diameter and entrapment efficiency of formulations, it is possible that a lower concentration of CS resulted in a lower encapsulation efficiency. Figure 6I displays the results, which agree with a previous study [22]. Increasing the amount of CS in the coagulation solution causes a denser membrane to form because more alginate–CS ionic connections are formed, making the gel structure less porous and increasing the drug entrapment. This may be due to the stronger and tighter structure of the CS-SALG gel beads, which will not allow the drug to diffuse from the beads during encapsulation.

### 2.6. Selection of the Optimized Formulation

**Figure 7:**
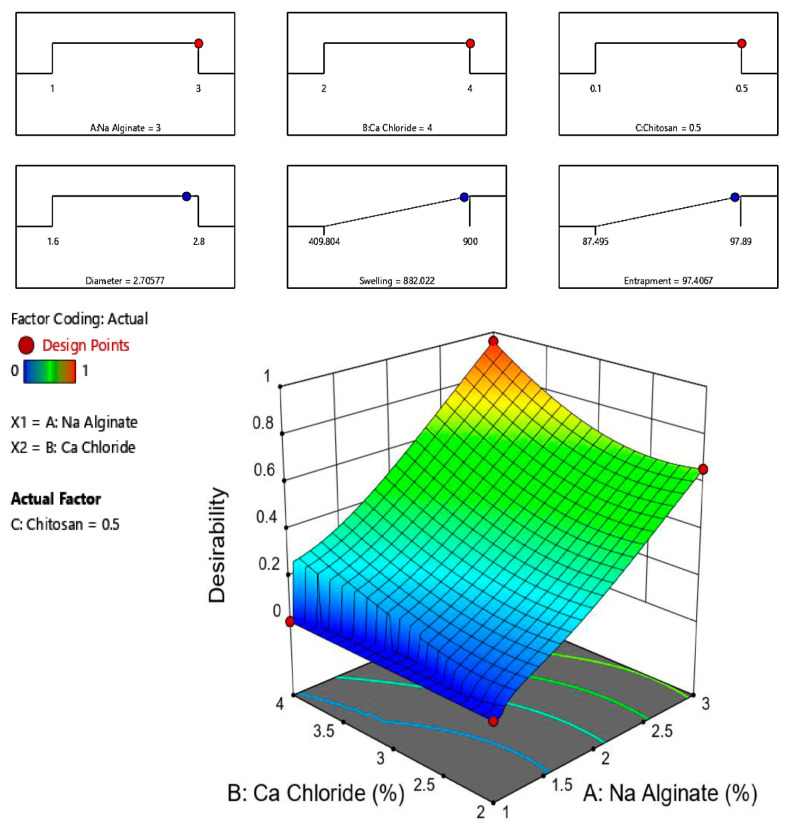
Desirability ramp of optimized conditions for bead processing conditions (**A**) and 3D response surface plot for the desirability function (**B**) for the optimal formulation #13 predicted by the Design-Expert software.

We chose the optimal formulation by the numerical optimization of Design-Expert^®^ software. Variable parameters were set within range. The optimized formulation in this study needed to have maximum entrapment, swelling, and diameter within range. F13 (Table 3) was chosen as the optimized formulation with a desirability of 0.959. The predicted SALG (X1 = 3% *w*/*v*), CaCl_2_ (X2 = 4% *w*/*v*), and polymer CS (X3 = 0.5%) values were obtained. These values predict a 2.706 mm diameter, 882.022% swelling and 97.424% EE. The optimized formulation was further characterized to confirm the validity and was found to be within limits (Figure 7A,B).

### 2.7. Morphology of Optimized Formula-Coated Beads

**Figure 8:**
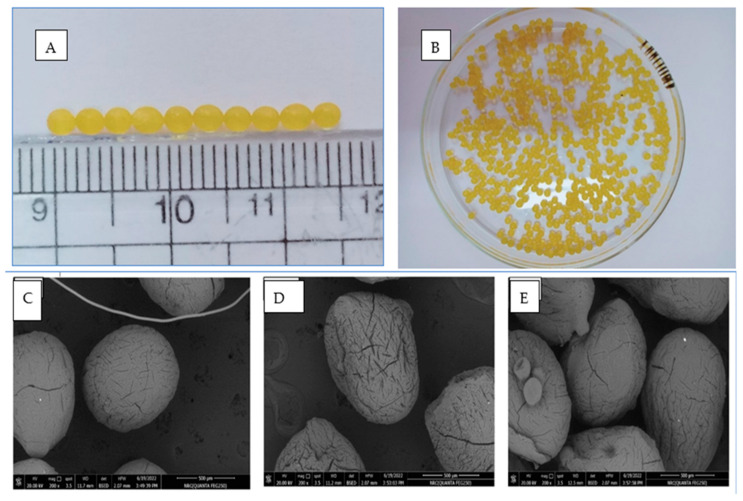
Digital photos of optimized formulation hydrogel beads, (**A**,**B**); particle size image scanning electron micrograph of optimized formulation hydrogel beads: (**C**) blank beads, uncoated; (**D**) loaded beads, uncoated; (**E**) loaded beads, Eudragit S 100–coated.

The surfaces of the beads were smooth; this led to a clear prolongation of the release time [23]. When the beads were wet, their shape was round, and their surface was smooth, as shown in Figure 8. Their diameter was about 2.6 mm. Images displaying the SEM analysis of blank, unloaded, drug-loaded, uncoated beads and drug-loaded Eudragit^®^ S-100-coated hydrogel beads can be seen in Figure 8C–E. The microstructure results from the polymeric network show that they are partially collapsing, resulting in fissures on their surfaces. Polymeric beads tend to crack when they dry out in the air. Figure 8C shows SEM micrographs of blank, uncoated beads, which had smoother surfaces and smaller sizes compared to the drug-loaded, uncoated beads (Figure 8D). The drug-loaded beads was shown to have a regular surface morphology. SEM images of the Eudragit^®^ S-100-coated hydrogel beads are given in Figure 8E. The enteric-coated hydrogel beads had smooth surfaces, in contrast to the uncoated beads, which possessed drug crystals. The SEM results published previously by one research group [24].revealed comparable findings throughout their experiment using Eudragit^®^ S-100-coated CS microspheres. As shown in Figure 8E, the drug-loaded, coated beads were spherical in shape.

### 2.8. FTIR for Optimized Formula-Coated Beads

Infrared spectroscopic analyses of the drugs (MSZ-complex and CUR-complex), SALG, chitosan, Eudragit^®^ S-100, their physical mixes, and the optimized formula-coated beads were acquired (Supplementary information, Figure S1A–G, respectively). Both the beads’ physical mixes and the optimized formulas exhibited absorption bands corresponding to their individual ingredients. The peak concentrations of CUR and MSZ in the formulation were not very prominent due to their dilution with the matrix components, as well as the inclusion complex’s effect on their absorption band intensities. After being coated with Eudragit^®^ S-100, beads exhibited reduced absorbance intensity and changes in the positions of absorbance peaks. Hydrogen bonding between Eudragit^®^ S-100 and the beads provides a plausible explanation for these alterations. 

### 2.9. DSC for Optimized Formula-Coated Beads

DSC thermograms of the drugs (MSZ-complex and CUR-complex), SALG, chitosan, Eudragit^®^ S-100, the optimized form physical mixture, and the optimized prepared beads after drying were acquired (Supplementary Information, Figure S2A–G, respectively). The optimized formulation physical mixture (Supplementary Information, Figure S2F) demonstrated small endothermic peaks corresponding to MSZ and CUR inclusion complexes at 255.52 °C and 177.6 °C, respectively, within the endothermic events of the other ingredients. However, the dried formulated beads’ thermogram (Supplementary Information, Figure S2G) showed a broad melting peak of the polymer matrix at high temperatures without any indication of the endothermic events of either MSZ or CUR inclusion complexes. This indicated the solubilization of the inclusion complexes within the polymer matrix.

### 2.10. In-Vitro Release and Release Kinetic Study

**Figure 9:**
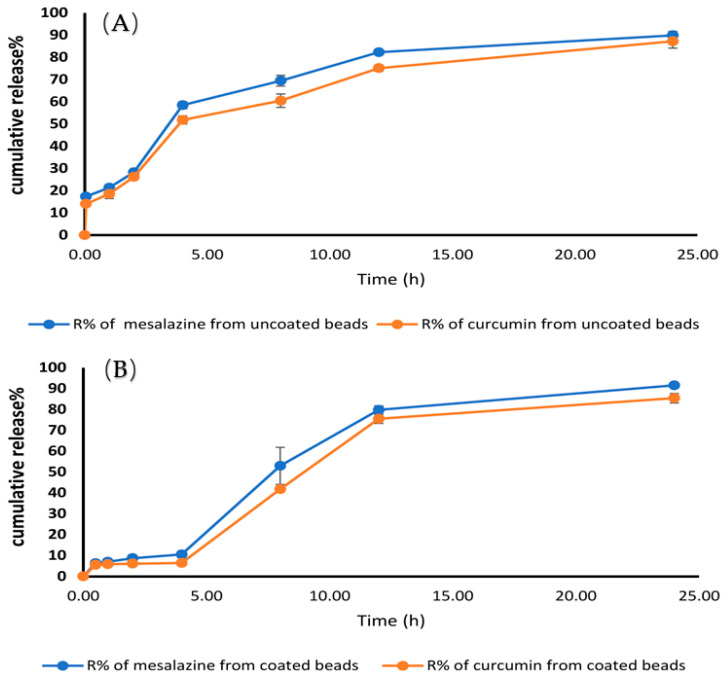
In-vitro release contours of the different ingredients. (**A**) Uncoated dual drug-loaded beads and (**B**) Eudragit-coated dual drug-loaded beads in pH variable buffers.

Figure 9 shows the in-vitro release characteristics of both uncoated and coated drug-loaded beads in a range of buffers with a decreasing pH. In the case of uncoated beads, 51.78 ± 1.82% of CUR and 58.46 ± 1.59% of MSZ was released in the initial 4 h. This situation is unacceptable for medications that need to be released locally in the colon. The release of drugs was delayed until the pH was above 7.0 by coating the beads with Eudragit^®^ S-100 polymer. For this experiment, Eudragit^®^ S-100 was used to cover the beads. Hydrogel beads encapsulating the drugs were shown to release their contents into simulated GIT fluids. Although the coated hydrogel beads showed promise, their initial drug release was disappointing. After 2 h, a minor fraction of the medicines could be detected in the pH 1.2 media. Only 6.01 ± 0.04% CUR and 8.64 ± 0.7% MSZ were released after 2 h, and 6.36 ± 0.11% CUR and 10.45 ± 1.52% MSZ were released after 4 h at pH 6.8. After 8 h, approximately 85.34 ± 2.3% CUR and 91.5 ± 1.2% MSZ were released at pH 7.4. Since the carboxyl groups in the Eudragit^®^ S-100 polymerize from neutral to alkaline conditions, our results show that the medication was sheltered from gastric acid and enzymes. Ionization compromises the film’s structural integrity, allowing the drug to escape. The membrane layer on the hydrogel beads was dissolved at pH 7.4. The medication was released once the polymer matrix swelled and was eroded.

CUR/MSZ combination-loaded beads protected against acetic acid induced ulcerative colitis.

### 2.11. Stability Study

Beads of the optimized formula showed very little change in size through five successive weeks at room temperature (Table 3).

**Table 3:** The average diameters of beads of optimized formula through five successive weeks.

Week No.	Diameter (mm)
Week 1	0.406 ± 0.011
Week 2	0.406 ± 0.005
Week 3	0.403 ± 0.006
Week 4	0.406 ± 0.005
Week 5	0.403 ± 0.005

### 2.12. Colon/Body Weight (C/B) Ratio

**Figure 10:**
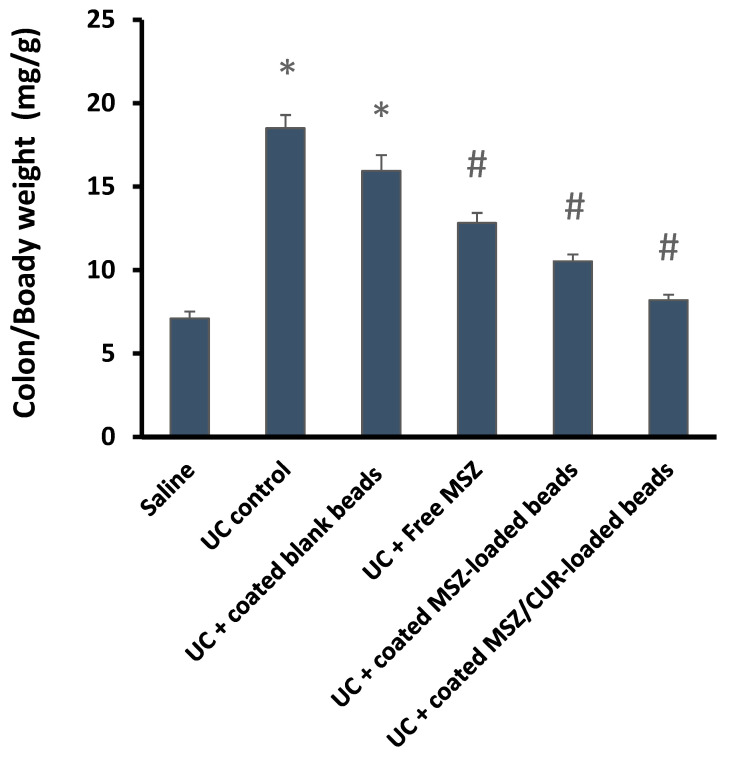
The colon/body weight ratio in rats exposed to acetic acid-induced colitis. *: versus Saline, #: versus UC control at *p* < 0.05.

Figure 10 displays the C/B ratio in rats with ulcerative colitis after oral administration of free MSZ medication, MSZ-complex-loaded beads, and CUR/MSZ-coated beads. The C/B ratio was significantly higher in the UC control rats (untreated animals) at 18.5 mg/g compared to the saline-treated rats (7.1 mg/g). The C/B ratio was 15.97 mg/g in rats treated with UC + blank coated beads, indicating that the beads had no discernible therapeutic impact and that significant inflammation in the colon persisted despite the treatment. The C/B ratios in rats treated with UC + free MSZ, UC + coated MSZ-loaded beads, and UC + coated CUR//MSZ-loaded beads were 12.84 mg/g, 10.53 mg/g, and 8.12 mg/g, respectively. All three of these groups experienced significant declines in the C/B ratio. Based on these findings, it seems that the coated beads hindered rapid drug delivery in the upper part of the GIT and that MSZ and CUR were released at the inflamed colon after the Eudragit^®^ S-100 had entirely dissolved at pH 7, which was achieved at the terminal ileum. Moreover, the C/B ratio of rats given CUR/MSZ-loaded beads was much lower than that of rats given free MSZ or coated MSZ-loaded beads. These results suggested that the simultaneous release of the two medications in the inflamed colonic region might have additive, synergistic effects on the colitis rats, implying that the loading of dual drugs in beads has the potential to reduce the colonic inflammation.

### 2.13. Evaluation of Colonic Macroscopic Injury

**Figure 11:**
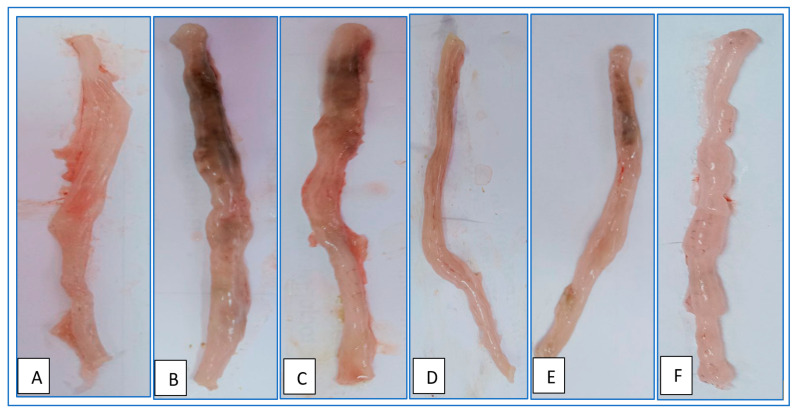
Photographs of the colons of the experimental groups. (**A**) Saline, (**B**) UC control (untreated group), (**C**) UC + blank beads, (**D**) UC + free CUR/MSZ, (**E**) UC + coated MSZ-loaded beads, and (**F**) UC + coated CUR/MSZ-loaded beads group. UC: ulcerative colitis.

Macroscopic colonography was a useful tool for tracking ulcerative colitis’s progression. The level of colon inflammation may be visually evaluated, and the success of treatment can be evaluated in direct correlation with the true inflammatory status of the colon. Representative images for each experimental group are shown in Figure 11. The colons of healthy rats were pink and unharmed on a macroscopic level (Figure 11A), while those taken from rats with colitis displayed signs of intestinal injury such as oedematose inflammation, ulcer development, hyperemia, and rigidity of the intestinal wall (Figure 11B). Figure 11C shows that inflammatory symptoms do not significantly improve in rats given blank beads. Macroscopically observable signs of colitis, such as colon thickness, necrosis, hyperemia, and ulceration, were present in all of the rats in this study group. However, in comparison to the animals in the UC control group, the rats treated with both free MSZ and coated MSZ-loaded beads showed significantly less localized inflammation and a smaller necrotic zone (Figure 11D,E).

Moreover, coated dual medication-laden beads significantly reduced inflammation levels. The colons of these rats showed mild necrotic changes and hyperemia of the colon. Very little difference could be seen between the colons of these rats and those of healthy rats, save for the absence of inflammation (Figure 11F). These findings corroborated that the oral delivery of coated beads significantly reduced colon inflammation, previously shown using the C/B ratio. In UC control rats, it was found that coated dual-loaded beads best took advantage of synergistic effects.

### 2.14. Histopathological Evaluation of Colon Specimens Stained with Haematoxylin and Eosin

**Figure 12:**
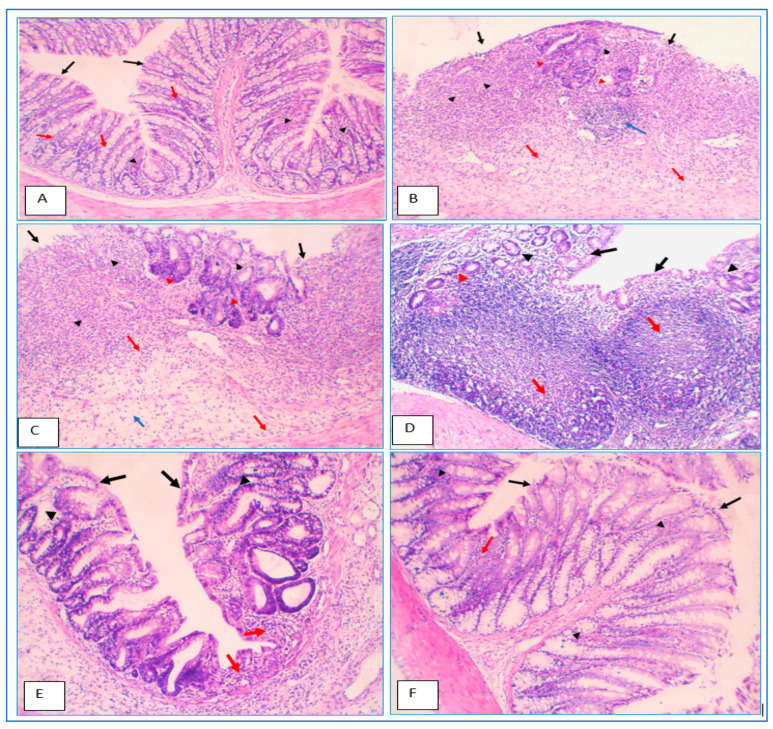
Histopathology of colon specimens in the study groups. (**A**) Saline, (**B**) UC control (untreated group), (**C**) UC + blank beads, (**D**) UC + free MSZ, (**E**) UC + coated MSZ-loaded beads, and (**F**) UC + coated CUR/MSZ-loaded beads group. Ulceration in surface epithelium (black arrows), filling of the whole lamina propria (black arrowheads), extension into the submucosa (red arrows), lymphoid aggregates (blue arrow), inflammatory cells attacking glands (red arrowhead), and areas of oedema (blue arrows) are present.

The findings from the C/B ratio and macroscopic observation may be supported by histological assessment. The rats in the healthy group had normal colon anatomy, as shown in Table 4 and Figure 12, with normal enterocytes, muscular mucosae, goblet cells, and submucosa (Figure 12A). Meanwhile, in the UC control group, the rats’ colons demonstrated mucosal injury and necrosis of granulation tissue, the destruction of the colon’s normal architecture, damage to enterocytes and goblet cells, and extensive mucosal and submucosal inflammatory infiltration (Figure 12B). The colons of rats treated with blank beads matched those observed in the UC control group (Figure 12C). In comparison with the UC control group, animals treated with free MSZ and coated MSZ-loaded beads demonstrated significant repair of goblet cells, relief of mucosal structures, and minor inflammatory infiltration (Figure 12D,E). Rats treated with coated dual drug-loaded beads had mostly normal mucosal architecture and repaired epithelial and goblet cells, with just a small amount of inflammatory infiltration through submucosa (Figure 12F). Histological analysis corroborated the efficacy of the coated dual medication in beads in treating colitis in rats by gradually releasing MSZ and CUR into the inflamed colon. In sum, the data presented above support the idea that the current formulated delivery system of coated dual medication-loaded beads to the colon may be useful in managing UC.

## 3. Conclusions

This article demonstrated, for the first time, the formulation of hydrogel beads containing MSZ and CUR complex coated with pH-sensitive polymers (Eudragit^®^ S-100) to provide a unique colonic delivery system of this combination. Beads were fabricated by ionotropic gelation method for the extended release of a MSZ-CUR combination. FTIR confirmed the interaction between drug molecules and polymer network. We confirmed the chemical stability and molecular dispersion of MSZ and CUR in beads. SEM studies confirmed the presence of pH-sensitive polymers (Eudragit^®^ S-100) on the outer surface of beads. The effect of the incorporation of CaCl_2_ on the cross-linking of SA chains, swelling studies, and drug release studies were thoroughly examined. The optimized formula F#13 showed the highest uptake of water at pH 7.4 compared to other formulations due to the formation of a less rigid network at a low concentration of SALG 1%. 

The drug release profiles at pH 7.4 were correlated with the highest swelling results. In-vitro release results for uncoated MSZ-CUR beads in acidic media after 4 h showed the highest drug release % of MSZ and CUR compared to MSZ-CUR-coated beads, which showed less drug release % of MSZ and CUR in the same media after 4 h. These results reflect the release of the majority of two drugs from the coated beads at colon rather than the stomach; hence, these beads possessed controlled drug delivery features. This colon-specific delivery system was able to deliver MSZ and CUR specifically to the colon site and thus significantly reduced the inflammatory reactions and alleviated pathological features in the rats suffering from ulcerative colitis, compared with MSZ only,. Consequently, this technique of drug delivery should be studied further in other rat inflammatory colon conditions. This formulation may prove useful in the clinical management of human ulcerative colitis.

## 4. Materials and Methods

### 4.1. Chemicals and Reagents

All chemicals were purchased in the analytical grades. Mesalamine, curcumin, SALG, chitosan, hydroxypropyl-β-cyclodextrin (HP-β-CD), and Eudragit^®^ S-100 were obtained from Sigma-Aldrich (St. Louis, MO, USA). Tri-ethyl citrate (TEC), liquid paraffin, span-80, acetic acid (99%), and sodium hydroxide were purchased from Sigma-Aldrich. Acetone, n-hexane, HCl, Mg-stearate, potassium dihydrogen phosphate, di-sodium-hydrogen-phosphate, methanol, and ethyl alcohol (99%) were procured from El-Nasr Pharmaceutical Co. for Chemicals (Cairo, Egypt). Anhydrous calcium chloride (Fine GRG 90%) was supplied by Fisher Scientific (Fairlawn, NJ, USA).

### 4.2. Phase Solubility Study

The complex stability and stoichiometry constant were determined using the phase solubility approach. Excess quantities of either CUR or MSZ (~30 mg) were transferred to aqueous solutions (each solution was 10 mL) containing different HP-β-CD concentrations (0–10 mM). We irradiated the prepared mixtures in an ultrasonic water bath for 30 min, then left them to sit at 25 °C for 5 days. When an equilibrium had been established, the supernatant solutions were diluted, filtered through a 0.45-µm filter, and then analyzed at 306 and 421 nm using an ultraviolet (UV) spectrophotometer, the Shimadzu UVPC UV-Vis spectrophotometer (Shimadzu, Japan) to determine the concentrations of MSZ and CUR, respectively, using the appropriate UV standard curve for each compound. We performed triplicate experiments and plotted curves between the concentration of HP-β-CD (x-axis) and concentrations of CUR or MSZ (y-axis). We determined the complexation constants following the technique documented previously [12].

### 4.3. Preparing and Characterizing the Drug/HP-β-CD Inclusion Complexes

The inclusion complex of CUR/HP-β-CD was formulated using CUR and HP-β-CD at a molar ratio of 1:1. HP-β-CD powder (1.54 g) was transferred into a 50-mL glass beaker followed by the addition of 5 mL of ethyl alcohol, and the mixture was gently stirred until the solubilization of HP-β-CD occurred. Then, 0.368 g of CUR was added to the previous solution and stirred until a transparent solution was obtained. The resulting solution was situated in a fume hood at 25 ± 1 °C for 24 h to slowly evaporate the solvent. The obtained solid residue was sieved through an 80-mesh sieve and preserved in the dark in a desiccator at 25 ± 1 °C [25].

Meanwhile, MSZ powder (0.153 g) was dissolved in a 2:1 (*v*/*v*) acetone/water mixture at room temperature with constant magnetic stirring. The resulting MSZ solution was added dropwise to another solution of 1.54 g HP-β-CD in water to formulate the MSZ/HP-β-CD inclusion complex with a 1:1 molar ratio [26]. The obtained solution was stirred with a magnetic stirrer at 40 °C for 5 h then heated to 60 °C until the solvent had completely evaporated. The obtained solid residue was sieved through an 80-mesh sieve and the final product was preserved in a desiccator at 25 ± 1 °C.

In order to attain characterization of the drug/HP-β-CD complex, a previous phase solubility study highlighted that complexes of 1:1 stoichiometry between CUR or MSZ and HP-β-CD are soluble [27]. Fourier-transform infrared (FTIR) spectroscopy and differential scanning calorimetry (DSC) were used to validate the formation of inclusion complexes. FTIR spectroscopy and DSC categorize the solid state of the drug/HP-β-CD complex [26].FTIR spectra were obtained using a Nicolet 6700 FT-IR spectrometer (ThermoFisher Scientific, Waltham, MA, USA) through a scanning scope ranging from 4000 to 400 cm^−1^. 

### 4.4. Formulation of CS/SALG Hydrogel Beads

Beads were formulated based on previously published procedures [28,29,30] with some modifications. A solution of 3% *w*/*v* SALG in distilled water was prepared by mechanical mixing at 900 rpm at room temperature. The solution was then heated to 70 °C. Amounts of inclusion complexes equivalent to 15 mg of MSZ and 15 mg of CUR were first dispersed into small volumes of distilled water, then mixed with the heated SALG solution at 900 rpm to form SALG-Complex mixture. SALG-Complex mixture was then extruded at flow rate of 1 mL. min^−1^ into a coagulation fluid with mechanical stirring at 200 rpm. The coagulation fluid was composed of an aqueous solution of 0.5% CS, 4% CaCl_2_, and 1% *v*/*v* acetic acid adjusted to be within a pH range of 5.0 ± 0.1. The beads were kept in the coagulation fluid for 30 min while stirring. The beads were then cleaned, air-dried, and stored for further experiments [31,32]. 

#### 4.4.1. Optimization of Hydrogel Bead Formulation Variables

The D-optimal design optimized the conditions for preparing CS/SALG beads. We used Design-Expert^®^ (version 11.1.2.0, Stat-Ease Inc., Minneapolis, MN, USA) to explore quadratic response surfaces and construct second-order polynomial models. Table 5 shows the independent and dependent variables. The experimental design was used in an effective model to analyze the relation between the independent variables and the responses and interactions. The experimental design included three variables and three responses. Encapsulation efficiency, swelling degree, and particle size were the measured dependent response factor variables. However, the independent variables were the SALG concentration (X1), CaCl_2_ concentration (X2) and CS concentration (X3; Table 5). Depending on preliminary research trials and data from the literature, the formulation variables and the maximum and minimum degrees of variable were set. A series of three sets of center-point repetitions were performed as a means of gauging experimental error. Table 5 displays the design matrix in its coded form.

#### 4.4.2. Characteristics of the Hydrogel Beads

##### Bead Diameter Analysis

The diameters of the beads were measured using a vernier caliper micrometer screw gauge, and average readings were calculated using at least 10 beads [33].

##### Swelling Experiments

The immersion technique was utilized to study the degree of bead swelling [20]. Swelling studies were conducted in a shaking water bath at 37 ± 0.5 °C. After weighing the dry beads, they were rehydrated using phosphate buffer solution at pH 7.4. Every two hours, the beads were taken out, blotted using filter papers to eliminate the excess amounts of the buffer, and the weight of the beads was recorded until they reached equilibrium. We calculated the degree of swelling by applying Equation (1) [34]:

(1) $$\%\;\mathrm{Swelling}=\frac{\mathrm{Wt}.\;\mathrm{of}\;\mathrm{swollen}\;\mathrm{beads}-\mathrm{Wt}.\;\mathrm{of}\;\mathrm{dry}\;\mathrm{beads}}{\mathrm{Wt}.\;\mathrm{of}\;\mathrm{dry}\;\mathrm{beads}}\times 100$$

##### Encapsulation Efficiency (EE%)

The amount of the drugs contained within the beads was calculated using a straightforward method. Hydrogel beads weighing 200 mg were precisely weighed and then crushed for complete drug extraction. The crushed beads were added to the methanol, and the mixture was agitated for a full day at room temperature. The fluid was filtered using a 0.45 µm Millipore^®^ syringe filter. The drug concentrations were measured at 306 and 421 nm for MSZ and CUR, respectively, using an ultraviolet (UV) spectrophotometer, the Shimadzu UVPC UV-Vis spectrophotometer (Shimadzu, Japan) and expressed as AQ. The drug encapsulation efficiency was calculated using Equation (2) [35].

(2) $$\mathrm{Drug}\;\mathrm{encapsulation}\;\mathrm{efficiency}\;\left(\mathrm{EE}\right)\%\;=\frac{\mathrm{AQ}}{\mathrm{TQ}}\times 100$$

where TQ is the theoretical drug concentration expected to be present in the tested quantity of the beads based on input quantity during the beads’ preparation. The measurements were conducted in triplicate.

#### 4.4.3. Optimization Employing the D-Optimal Design

The optimal formula was selected depending on the response variables’ desirability, and it was tested physiochemically and in vitro. Formula #13 was selected as the best formula.

### 4.5. Enteric Coating of the Selected Optimal Hydrogel Bead Formula #13

The optimized formula of CUR/MSZ hydrogel beads (#13) was coated using S- Eudragit^®^ S-100, a colon-targeting, pH-dependent polymer. To coat the formulated beads, we utilized the oil/oil solvent evaporation method. During coating, the dispersion phase was Eudragit^®^ S-100 (5% *m*/*v*) dissolved in a 2:1 acetone/ethanol mixture, and we then added the pre-formulated hydrogel beads over 4 h. For the emulsion’s dispersing phase, we used 70 mL of light liquid paraffin and 2% span 80 and stirred them for 3 h at 40 °C (at 1000 rpm). We then filtered the coated beads, rinsed them with n-hexane, sprinkled them with Mg-stearate, and left them to dry at room temperature [36].We then kept them in airtight containers until we began the enteric coating process.

#### 4.5.1. Scanning Electron Microscopy Analysis of the Optimized Formula #13 of the Coated Beads

The surface and morphologies of hydrogel beads were inspected using Scanning Electron Microscopy (SEM) (JEOL, JSM-6380LV, Akishima Tokyo, Japan) [2].We fixed the samples to aluminum rods using double-sided tape and coated them with gold sputtering, then scanned at 30 kV.

#### 4.5.2. FTIR Analysis of the Optimized Formula-Coated Beads

Before submitting samples to FTIR analysis, beads were ground into a powder using a mortar and pestle. The material was then placed under an FTIR probe with a range of 4000 to 400 cm^−1^ and 95% transmittance. FTIR spectroscopy was used to detect the presence of interactions and identify changes in functional group composition.

#### 4.5.3. DSC Studies for the Optimized Formula of the Coated Beads

Thermograms of DSC measurements were taken with DSC 60 Software (60TA-60WS). The phase equilibria, polymorphic transitions, melting, crystallization, and decomposition behaviors of the beads were characterized using DSC analyses. For the DSC procedure, the weight of the dried beads ranged from 3 mg to 7 mg. The studies were carried out in aluminum pans that had been sealed with a cover containing pin holes. A nitrogen flow was used to evaluate the sample at a scan rate of 10 °C per min throughout a temperature range of 25–300 °C. The DSC measured melting point, crystallinity changes, and the presence or absence of drug-carrier-adsorbent interactions.

#### 4.5.4. In-Vitro Drug Release from the Optimized Formula of the Coated Beads

We conducted the drug release experiment in 0.1 N HCl with a pH value equaling 1.2 at 100 rpm for 120 min in a USP apparatus type II (Copley, England) to mimic the conditions in the stomach. For another 2 h, the drug release investigation was conducted in phosphate buffer to simulate the small intestine environment (pH 6.8). A pH 7.4 phosphate solution was utilized for an additional 20 h to simulate the colon. At predefined time intervals until 24 h, 5-mL aliquots were removed and substituted by an equivalent volume of new buffer. All the samples’ medication concentrations were measured at 306 and 421 nm for MSZ and CUR, respectively, using an ultraviolet (UV) spectrophotometer, the Shimadzu UVPC UV-Vis spectrophotometer (Shimadzu, Japan).

### 4.6. Stability Study

The sizes of beads of the optimized formula (F 13) were measured at room temperature using a graduated ruler as a factor to evaluate the physical stability of the beads. The sizes were measured through five successive weeks and the average readings were taken for at least 10 beads. Measurements were conducted in triplicate for the five successive weeks [37].

### 4.7. In-Vivo Study of the Protective Effect of the Optimized Formula-Coated Beads

Male Wistar albino rats were obtained from the animal house at Suez Canal University. The baseline body weight was 170–200 g. We allocated the rats to five experimental groups of five animals each. They were housed at constant relative humidity and temperature and with a normal light-dark cycle, with access to water and commercial pellets ad libitum. Ethics committee at the Faculty of Pharmacy, Suez Canal University, Ismailia, Egypt approved the study protocol in September 2022 (202009PHDA1). The rats were fasted for 24 h before being given colitis-inducing chemicals.

#### 4.7.1. Induction of Ulcerative Colitis

Ulcerative colitis was induced in rats by injecting 1 mL of acetic acid (4% *v*/*v*) via a soft 6-Fr pediatric catheter into the colon anus (intra-rectal route) under mild anesthesia (sodium thiopental) [38]. The anus was catheterized up to 6 cm, and the dose of acetic acid was injected. Air was supplied at a rate of 2 mL/min just before catheter removal to ensure that acetic acid was distributed evenly throughout the colon. Rectal hemorrhage and loose feces were seen in the rats, verifying the induction of ulcerative colitis. After 2 days of confinement without treatment, a fully-fledged ulcerative colitis model was developed in the rats.

#### 4.7.2. Experimental Animal Groups

The drug-loaded beads were administered to the groups two days after induction of ulcerative colitis. For the next eight days, the rats received a single dose daily. They were randomly divided into six groups:

(1): Saline control: received saline solution (parallel to acetic acid application), (2) UC control, (3) UC + blank beads, (4) UC + free MSZ (equivalent to 20 mg/kg; administered orally), (5) UC + coated MSZ beads (equivalent to 20 mg/kg; administered orally), (6) UC + optimized formulation beads of CUR/MSZ coated with Eudragit^®^ S-100 (curcumin 20 mg/kg). 

#### 4.7.3. Scarification of Rats and Collection of Colon Specimens

Animals were weighed soon before treatment and just before autopsy to see if colitis affected body weight. The animals were slaughtered 24 h after the last medication delivery. We then dissected the full length of the colon and measured its length. After cutting the colon in half lengthwise, rinsing it in cold, normal saline, removing the fat and mesentery, and then blotting the intestines dry with filter paper, the weight was recorded. Colon oedema and inflammatory severity were evaluated using the colon mass index, which was estimated by the division of the colon weight in milligrams by the total body weight in grams. Visual evaluation of ulcer projections was performed, and the specimen was fixed in 10% *v*/*v* formalin solution and stored for histological examination [39].

#### 4.7.4. Calculation of the Colon/Body Weight Ratio

Luminal content was removed from weighted samples of the colon by opening the samples longitudinally and rinsing them with cold saline solution. Rats’ colon-to-body weight ratios were determined by dividing the colon weight by the overall rat body weight [40].

#### 4.7.5. Histological Procedures and Assessment of the Colon Specimens

Colonic damage was measured via histological analysis. The colon specimens were collected from the distal colon and submerged in 10% phosphate buffered formalin before being fixed in paraffin. Hematoxylin and eosin were used to stain sections of 4 um thickness. A pathologist examined the slides for epithelial damage, architectural alterations, mononuclear cell infiltration, and ulceration [41]. Colon tissue sections were tested for pathological developments and scored for the following [42]: inflammation [none (0)/slight (1)/moderate (2)/and severe (3)], inflamed area/extent [mucosa (1)/mucosa and submucosa (2)/and transmural (3)], crypt damage [none (0)/basal 1/3 damaged (1)/basal 2/3 damaged (2)/only the surface epithelium is intact (3)/and entire crypt and epithelium are lost (4)], and percent involvement [1–25% (1)/26–50% (2)/51–75% (3)/and 76–100% (4)].

#### 4.7.6. Statistical Analysis for the Results

Data have been denoted as the mean with standard deviation. We checked the difference between the study groups by applying one-way analysis of variance (ANOVA) and applying Bonferroni’s test for a post-hoc analysis at *p* < 0.05.

## Figures and Tables

**Table 1:** Phase-solubility data in water for CUR/HP-β-CD and MSZ/HP-β-CD systems at 37 °C.

Concentration of Cyclodextrin (mMol)	Solubility of CUR (mMol) with HP-β-CD	Solubility of MSZ (mMol) with HP-β-CD
0	0.196 ± 0.013	0.833 ± 0.045
1	0.442 ± 0.022	1.856 ± 0.081
2	0.768 ± 0.041	2.538 ± 0.095
3	1.074 ± 0.053	3.333 ± 0.137
4	1.420 ± 0.068	4.280 ± 0.231
5	1.739 ± 0.077	5.038 ± 0.256
6	2.158 ± 0.091	6.174 ± 0.343
7	2.564 ± 0.097	7.083 ± 0.375
8	2.910 ± 0.136	7.803 ± 0.387
9	3.269 ± 0.142	8.636 ± 0.401
10	3.609 ± 0.145	9.962 ± 0.434

Data are mean ± SD, *n* = 3.

**Table 4:** Histopathological evaluation of colon tissue sections.

Group	Histopathology Scoring
Saline control	Inflammation (1). Score = 1
UC control (untreated)	Inflammation (3), Inflamed area (3), Crypt damage (3), %involvement (3). Score = 12 *
UC + blank beads	Inflammation (3), Inflamed area (3), Crypt damage (3), %involvement (3). Score = 12 *
UC + free MSZ	Inflammation (2), Inflamed area (2), Crypt damage (2), %involvement (2). Score = 8 *
UC + coated MSZ-loaded beads	Inflammation (1), Inflamed area (1), Crypt damage (1), %involvement (1). Score = 4 #
UC + coated CUR/MSZ-loaded beads	Inflammation (1), Inflamed area/extent (1). Score = 2 #

*: versus Saline, #: versus UC control at *p* < 0.05. UC: ulcerative colitis.

**Table 5:** The independent variables and dependent variables used in the D-optimal design.

Factors	Independent Variables		Low (−1)	Medium (0)	High (+1)
X_1_	Na Alginate (SALG)	%	1	2	3
X_2_	Ca Chloride	%	2	3	4
X_3_	Chitosan (CS)	%	0.1	0.3	0.5
Response	Dependent Variables				
Y_1_	Diameter	Mm	In range		
Y_2_	Swelling	%	Maximize		
Y_3_	Entrapment Efficiency	%	Maximize		

## Data Availability

Data are available from Eman Heikal upon request.

## Supplementary Materials

The following supporting information can be downloaded at: https://www.mdpi.com/article/10.3390/gels9040264/s1, Figure S1: FT-IR spectra of the components of the complexes. (A) MSZ and HP-β-CD inclusion complex, (B) curcumin and HP-β-CD inclusion complex, (C) Na-alginate, (D) Chitosan, (E) Eudragit^®^ S-100, (F) Optimized form physical mixture and (G) Optimized beads formula. Figure S2. DSC spectra of the components of the complexes. (A) MSZ-HP-β-CD inclusion complex, (B) curcumin-HP-β-CD inclusion complex, (C) Na-alginate, (D) Chitosan, (E) Eudragit^®^ S-100, (F) Optimized form a physical mixture and (G) Optimized beads formula; Figure S3. (A) UV-Vis spectrum of beads plain formula with measurements at 306 and 421nm showing no absorbance. (B) Standard mixture of MSZ and CUR for comparison; Figure S4. (A) Linearity of MSZ UV-Vis Measurements at 306nm, (B) Linearity of CUR UV-Vis Measurements at 421 nm.

## References

[1] Göke K., Lorenz T., Repanas A., Schneider F., Steiner D., Baumann K., Bunjes H., Dietzel A., Finke J.H., Glasmacher B. (2018). Novel strategies for the formulation and processing of poorly water-soluble drugs. Eur. J. Pharm. Biopharm..

[2] Sookkasem A., Chatpun S., Yuenyongsawad S., Wiwattanapatapee R. (2015). Alginate beads for colon specific delivery of self-emulsifying curcumin. J. Drug Deliv. Sci. Technol..

[3] Kaplan G.G., Windsor J.W. (2021). The four epidemiological stages in the global evolution of inflammatory bowel disease. Nat. Rev. Gastroenterol. Hepatol..

[4] Sood A., Ahuja V., Midha V., Sinha S.K., Pai C.G., Kedia S., Mehta V., Bopanna S., Abraham P., Banerjee R. (2020). Colitis and Crohn’s Foundation (India) consensus statements on use of 5-aminosalicylic acid in inflammatory bowel disease. Intest. Res..

[5] Deshmukh R., Harwansh R.K., Das Paul S., Shukla R. (2020). Controlled release of sulfasalazine loaded amidated pectin microparticles through Eudragit S 100 coated capsule for management of inflammatory bowel disease. J. Drug. Deliv. Sci. Technol..

[6] Deshmukh R., Harwansh R.K. (2021). Preformulation Considerations Development and Evaluation of Mesalamine Loaded Polysaccharide-Based Complex Mucoadhesive Beads for Colon Targeting. Indian J. Pharm. Educ. Res..

[7] Seenan J.P., Lafferty H. (2019). Gastroenterologic Problems.

[8] Sood N., Bhardwaj A., Mehta S., Mehta A. (2016). Stimuli-responsive hydrogels in drug delivery and tissue engineering. Drug Deliv..

[9] Chifiriuc M.C., Mihai Grumezescu A., Grumezescu V., Bezirtzoglou E., Lazar V., Bolocan A. (2014). Biomedical applications of natural polymers for drug delivery. Curr. Org. Chem..

[10] Wu T., Yu S., Lin D., Wu Z., Xu J., Zhang J., Ding Z., Miao Y., Liu T., Chen T. (2020). Preparation, characterization, and release behavior of doxorubicin hydrochloride from dual cross-linked chitosan/alginate hydrogel beads. ACS Appl. Bio Mater..

[11] Ofridam F., Tarhini M., Lebaz N., Gagnière É., Mangin D., Elaissari A. (2021). pH-sensitive polymers: Classification and some fine potential applications. Polym. Adv. Technol..

[12] Lu Z., Cheng B., Hu Y., Zhang Y., Zou G. (2009). Complexation of resveratrol with cyclodextrins: Solubility and antioxidant activity. Food Chem..

[13] Mura P. (2015). Analytical techniques for characterization of cyclodextrin complexes in the solid state: A review. J. Pharm. Biomed. Anal..

[14] Elnashar M.M., Yassin M.A., Moneim A.E.-F.A., Bary E.M.A. (2010). Surprising performance of alginate beads for the release of low-molecular-weight drugs. J. Appl. Polym. Sci..

[15] Dai Y.N., Li P., Zhang J.P., Wang A.Q., Wei Q. (2008). Swelling characteristics and drug delivery properties of nifedipine-loaded pH sensitive alginate—Chitosan hydrogel beads. J. Biomed. Mater. Res. Part B Appl. Biomater..

[16] Malakar J., Nayak A.K. (2012). Formulation and statistical optimization of multiple-unit ibuprofen-loaded buoyant system using 23-factorial design. Chem. Eng. Res. Des..

[17] Kerdsakundee N., Mahattanadul S., Wiwattanapatapee R. (2015). Development and evaluation of gastroretentive raft forming systems incorporating curcumin-Eudragit^®^EPO solid dispersions for gastric ulcer treatment. Eur. J. Pharm. Biopharm..

[18] Liu Z., Jiao Y., Zhang Z. (2007). Calcium-carboxymethyl chitosan hydrogel beads for protein drug delivery system. J. Appl. Polym. Sci..

[19] Mennini N., Furlanetto S., Cirri M., Mura P. (2012). Quality by design approach for developing chitosan-Ca-alginate microspheres for colon delivery of celecoxib-hydroxypropyl-β-cyclodextrin-PVP complex. Eur. J. Pharm. Biopharm..

[20] Anal A.K., Stevens W.F. (2005). Chitosan—Alginate multilayer beads for controlled release of ampicillin. Int. J. Pharm..

[21] Sinha P., Udhumansha U., Rathnam G., Ganesh M., Jang H.T. (2018). Capecitabine encapsulated chitosan succinate-sodium alginate macromolecular complex beads for colon cancer targeted delivery: In vitro evaluation. Int. J. Biol. Macromol..

[22] Mi Y., Su R., Fan D.-D., Zhu X.-L., Zhang W.-N. (2013). Preparation of N, O-carboxymethyl chitosan coated alginate microcapsules and their application to Bifidobacterium longum BIOMA 5920. Mater. Sci. Eng. C.

[23] Martins A.F., Bueno P.V., Almeida E.A., Rodrigues F.H., Rubira A.F., Muniz E.C. (2013). Characterization of N-trimethyl chitosan/alginate complexes and curcumin release. Int. J. Biol. Macromol..

[24] Thakral N.K., Ray A.R., Majumdar D.K. (2010). Eudragit S-100 entrapped chitosan microspheres of valdecoxib for colon cancer. J. Mater. Sci. Mater. Med..

[25] Jantarat C., Sirathanarun P., Ratanapongsai S., Watcharakan P., Sunyapong S., Wadu A. (2014). Curcumin-hydroxypropyl-β-cyclodextrin inclusion complex preparation methods: Effect of common solvent evaporation, freeze drying, and pH shift on solubility and stability of curcumin. Trop. J. Pharm. Res..

[26] Tang P., Sun Q., Zhao L., Pu H., Yang H., Zhang S., Gan R., Gan N., Li H. (2018). Mesalazine/hydroxypropyl-$β$-cyclodextrin/chitosan nanoparticles with sustained release and enhanced anti-inflammation activity. Carbohydr. Polym..

[27] Tang P., Ma X., Wu D., Li S., Xu K., Tang B., Li H. (2016). Posaconazole/hydroxypropyl-β-cyclodextrin host—Guest system: Improving dissolution while maintaining antifungal activity. Carbohydr. Polym..

[28] Shi J., Alves N.M., Mano J.F. (2008). Chitosan coated alginate beads containing poly (N-isopropylacrylamide) for dual-stimuli-responsive drug release. J. Biomed. Mater. Res. Part B.

[29] Karakas C.Y., Ordu H.R., Bozkurt F., Karadag A. (2022). Electrosprayed chitosan-coated alginate—Pectin beads as potential system for colon-targeted delivery of ellagic acid. J. Sci. Food Agric..

[30] Padhmavathi V., Shruthy R., Preetha R. (2021). Chitosan coated skim milk-alginate microspheres for better survival of probiotics during gastrointestinal transit. J. Food. Sci. Technol..

[31] Sun X., Liu C., Omer A.M., Yang L.Y., Ouyang X.K. (2019). Dual-layered pH-sensitive alginate/chitosan/kappa-carrageenan microbeads for colon-targeted release of 5-fluorouracil. Int. J. Biol. Macromol..

[32] Gioumouxouzis C.I., Chatzitaki A.T., Karavasili C., Katsamenis O.L., Tzetzis D., Mystiridou E., Bouropoulos N., Fatouros D.G. (2018). Controlled release of 5-fluorouracil from alginate beads encapsulated in 3D printed pH-responsive solid dosage forms. AAPS PharmSciTech.

[33] Zeeb B., Saberi A.H., Weiss J., McClements D.J. (2015). Retention and release of oil-in-water emulsions from filled hydrogel beads composed of calcium alginate: Impact of emulsifier type and pH. Soft Matter..

[34] Omer A.M., Taher M.A., Hamed A.M., Ali A.M., Tamer T.M., Eldin M.S.M. (2019). Development of smart alginate/chitosan grafted microcapsules for colon site-specific drug delivery. Egypt J. Chem..

[35] Umaredkar A.A., Dangre P.V., Mahapatra D.K., Dhabarde D.M. (2020). Fabrication of chitosan-alginate polyelectrolyte complexed hydrogel for controlled release of cilnidipine: A statistical design approach. Mater. Technol..

[36] Rehman S., Ranjha N.M., Raza M.R., Hanif M., Majed A., Ameer N. (2021). Enteric-coated Ca-alginate hydrogel beads: A promising tool for colon targeted drug delivery system. Polym. Bull..

[37] Othman S.F.C., Samsudin M.N., Abdullah N.I.W. (2023). Assessing the Stability of Essential Oil Encapsulated in Hydrogel Beads: Assessing the Stability of Essential Oil Encapsulated in Hydrogel. J. Trop. Life Sci..

[38] Sawarkar S.P., Deshpande S.G., Bajaj A.N., Nikam V.S. (2015). In vivo evaluation of 5-ASA colon-specific tablets using experimental-induced colitis rat animal model. AAPS PharmSciTech.

[39] Singh A., Mandal U.K., Narang R.K. (2021). Development and characterization of enteric coated pectin pellets containing mesalamine and *Saccharomyces boulardii* for specific inflamed colon: In vitro and in vivo evaluation. J. Drug Deliv. Sci. Technol..

[40] Nidhi, Rashid M., Kaur V., Hallan S.S., Sharma S., Mishra N. (2016). Microparticles as controlled drug delivery carrier for the treatment of ulcerative colitis: A brief review. Saudi Pharm. J..

[41] Desai N., Momin M. (2020). Colon targeted bioadhesive pellets of curcumin and cyclosporine for improved management of inflammatory bowel disease. Drug Deliv. Transl. Res..

[42] Zhang Y., Zhao X., Zhu Y., Ma J., Ma H., Zhang H. (2018). Probiotic mixture protects dextran sulfate sodium-induced colitis by altering tight junction protein expressions and increasing Tregs. Mediat. Inflamm..

