# Supplementary material for: Development of Novel pH-Sensitive Eudragit Coated Beads Containing Curcumin-Mesalamine Combination for Colon-Specific Drug Delivery
Source: Gels. 2023 Mar 23;9(4):264. doi: 10.3390/gels9040264 (PMC10137603; doi:10.3390/gels9040264)
Supplement: Supplementary file 1 [file gels-09-00264-s001.zip › gels-2236558-supplementary.pdf]

Supplementary Information

Development of Novel pH-Sensitive Eudragit Coated Beads Containing Curcumin-Mesalamine Combination for Colon-Specific Drug Delivery

Eman J. Heikal [1,2], Rashad M. Kaoud[3], Shadeed Gad [1], Hatem I. Mokhtar[4], Abdullah Alattar[5], Reem Alshaman[5], Sawsan A. Zaitone[5,6], Yasser M. Moustafa[6,7] and Taha M. Hammady[1]

[1]Department of Pharmaceutics and Industrial Pharmacy, Faculty of Pharmacy, Suez Canal University, Ismailia, Egypt
[2]Faculty of Pharmacy, The University of Mashreq, Baghdad, Iraq
[3]Pharmacy Department, Ashur University College, PO Box 10047, Baghdad, Iraq
[4]Department of Pharmaceutical Chemistry, Faculty of Pharmacy, Sinai University-Kantara Branch, Ismailia 41636, Egypt
[5]Department of Pharmacology & Toxicology, Faculty of Pharmacy, University of Tabuk, Tabuk 71491, Saudi Arabia
[6]Department of Pharmacology and Toxicology, Faculty of Pharmacy, Suez Canal University, Ismailia 41522, Egypt. Sawsan_zaytoon@pharm.suez.edu.eg
[7]Department of Pharmacology and Toxicology, Faculty of Pharmacy, Badr University in Cairo, Cairo 11829, Egypt.
* Correspondence: author: shaded_abdelrahman@pharm.suez.edu.eg, Tel: 002-01003934422.

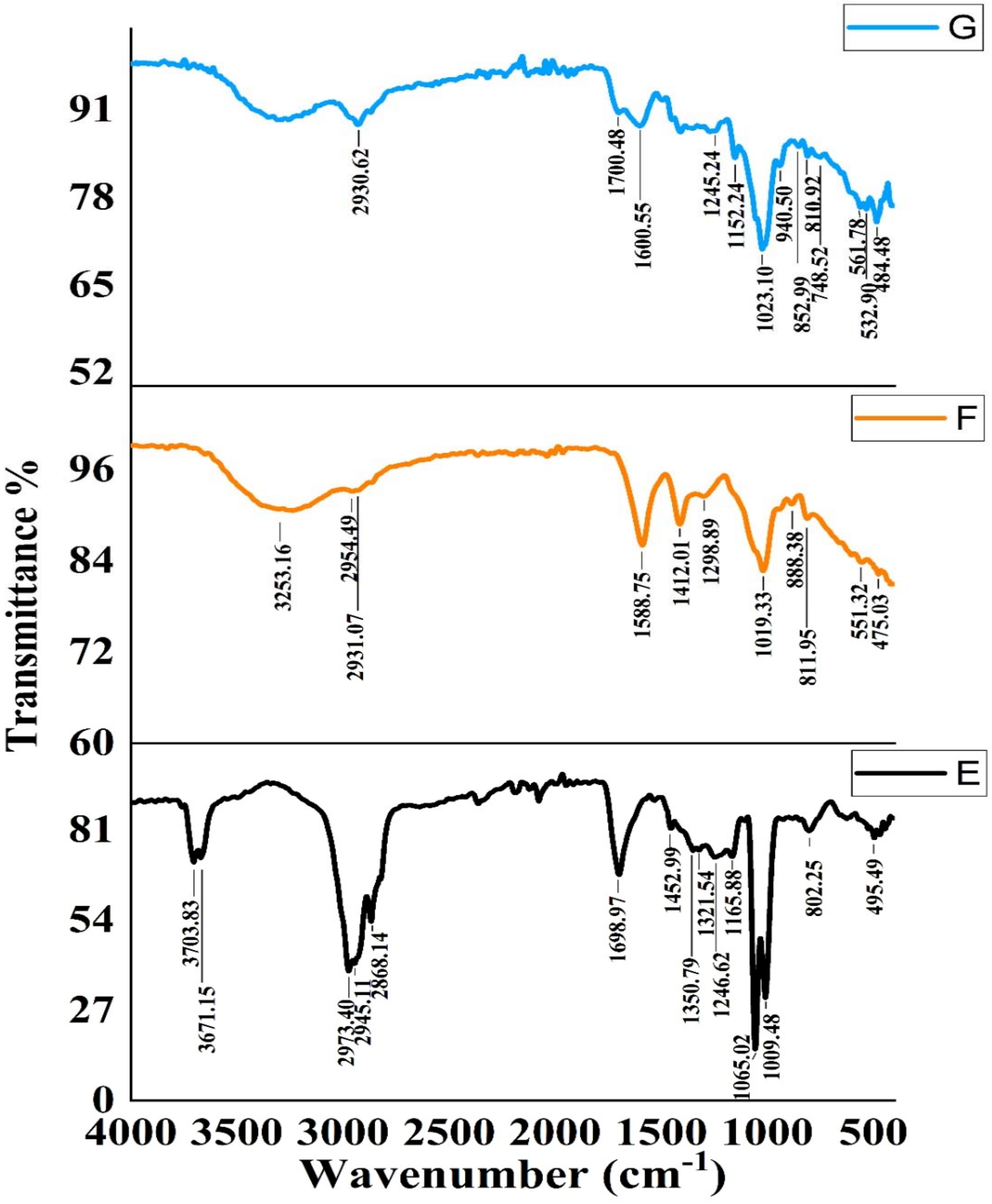
G
F
E
Transmittance %
Wavenumber (cm-1)
91
78
65
52
96
84
72
60
81
54
27
0
4000
3500
3000
2500
2000
1500
1000
500
2930.62
1700.48
1600.55
1245.24
1152.24
1023.10
940.50
852.99
810.92
748.52
561.78
532.90
484.48
3253.16
2954.49
2931.07
1588.75
1412.01
1298.89
1019.33
888.38
811.95
551.32
475.03
3703.83
3671.15
2973.40
2945.11
2868.14
1698.97
1452.99
1350.79
1321.54
1246.62
1165.88
1065.02
1009.48
802.25
495.49

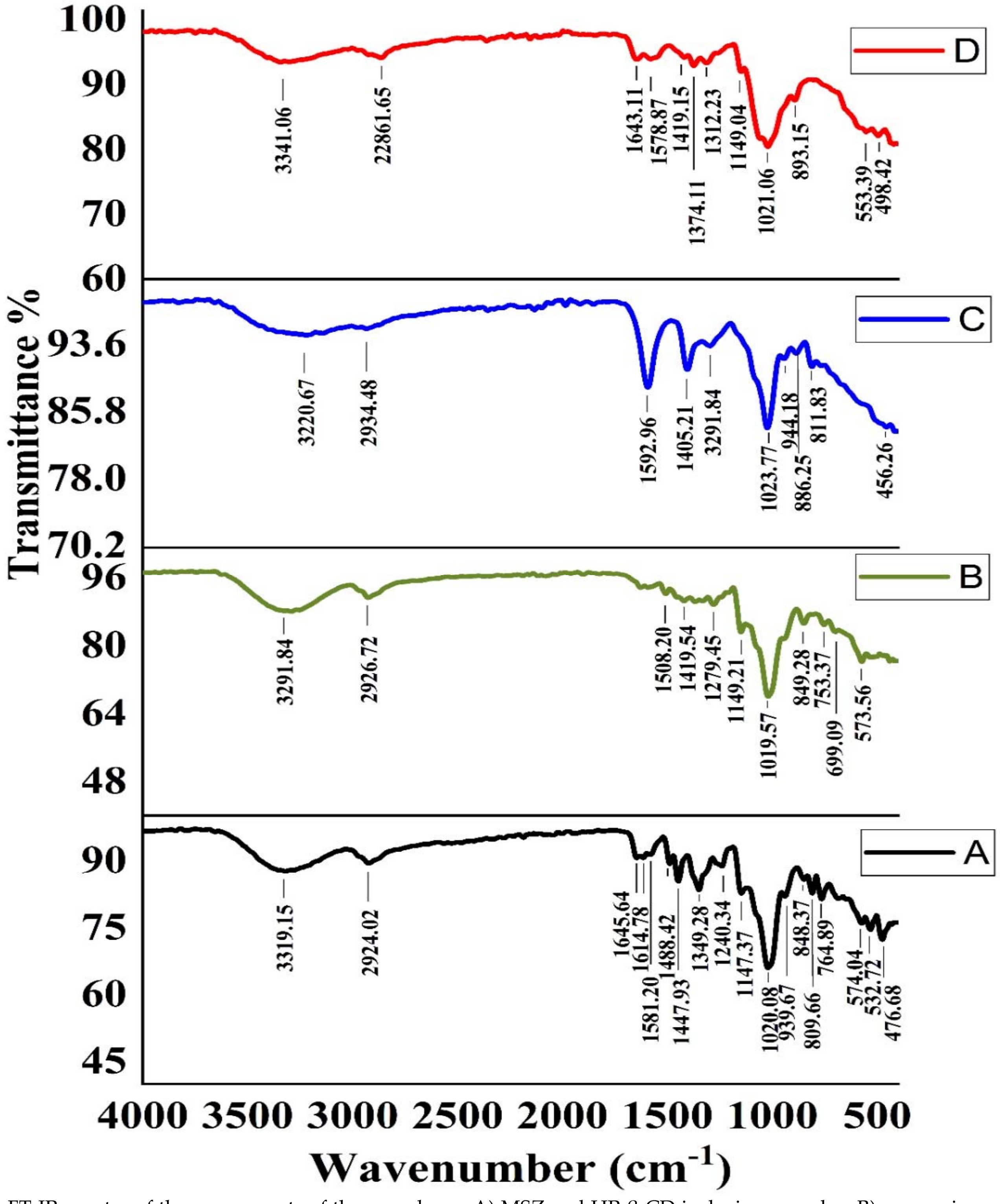

Figure S1 FT-IR spectra of the components of the complexes. A) MSZ and HP-β-CD inclusion complex, B) curcumin and HP-β-CD inclusion complex, C) Na-alginate, D) Chitosan, E) Eudragit® S-100, F) Optimized form physical mixture and G) Optimized beads formula.

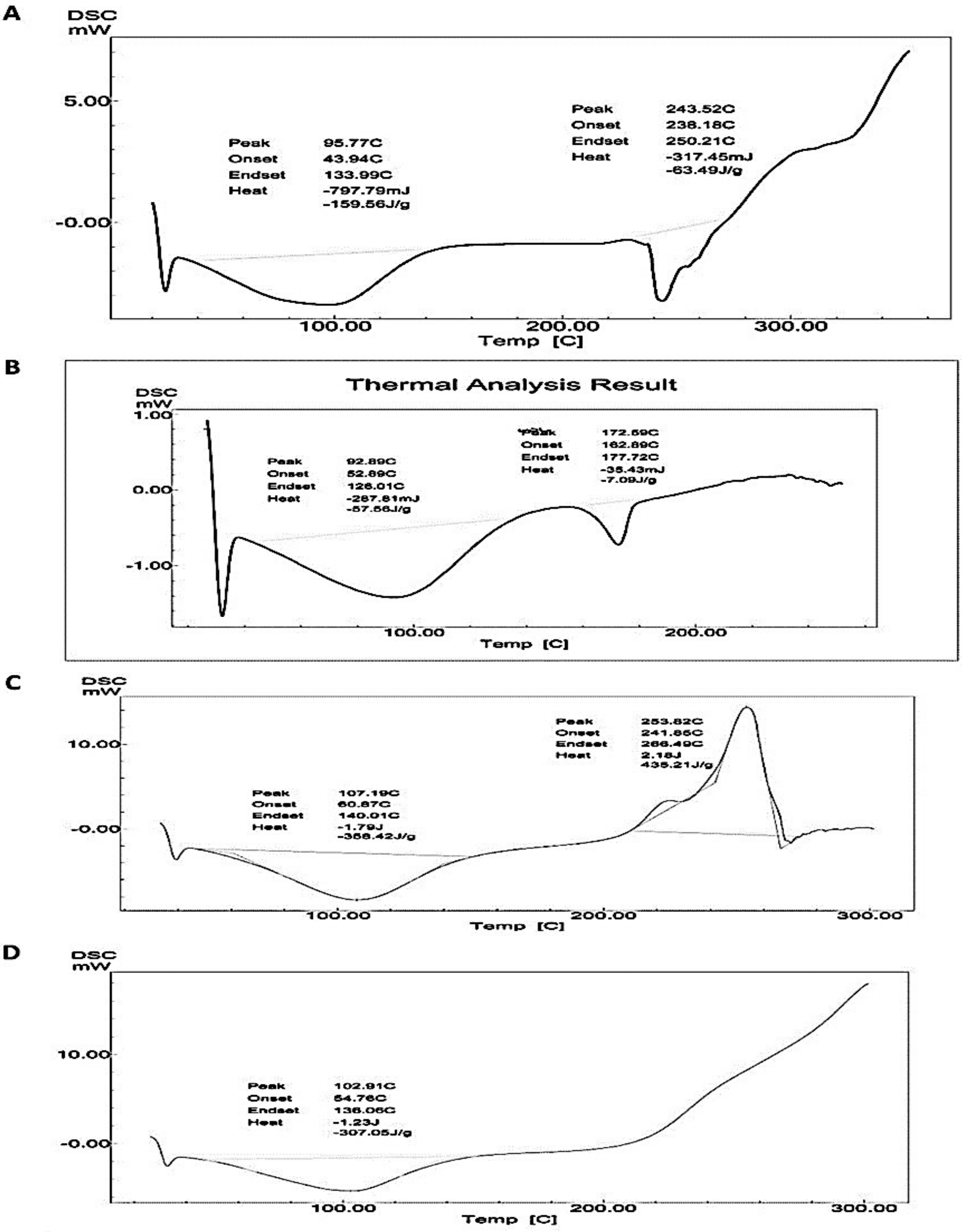
A
DSC
mW
5.00
-0.00
Peak 95.77C
Onset 43.94C
Endset 133.99C
Heat -797.79mJ
-159.56J/g
Peak 243.52C
Onset 238.18C
Endset 250.21C
Heat -317.45mJ
-63.49J/g
100.00
200.00
300.00
Temp [C]
B
Thermal Analysis Result
DSC
mW
1.00
0.00
-1.00
Peak 92.89C
Onset 52.89C
Endset 126.01C
Heat -287.81mJ
-57.56J/g
Peak 172.59C
Onset 162.89C
Endset 177.72C
Heat -35.43mJ
-7.09J/g
100.00
200.00
Temp [C]
C
DSC
mW
10.00
-0.00
Peak 107.19C
Onset 60.87C
Endset 140.01C
Heat -1.79J
-358.42J/g
Peak 253.82C
Onset 241.85C
Endset 266.49C
Heat 2.18J
435.21J/g
100.00
200.00
300.00
Temp [C]
D
DSC
mW
10.00
-0.00
Peak 102.91C
Onset 54.76C
Endset 136.06C
Heat -1.23J
-307.05J/g
100.00
200.00
300.00
Temp [C]

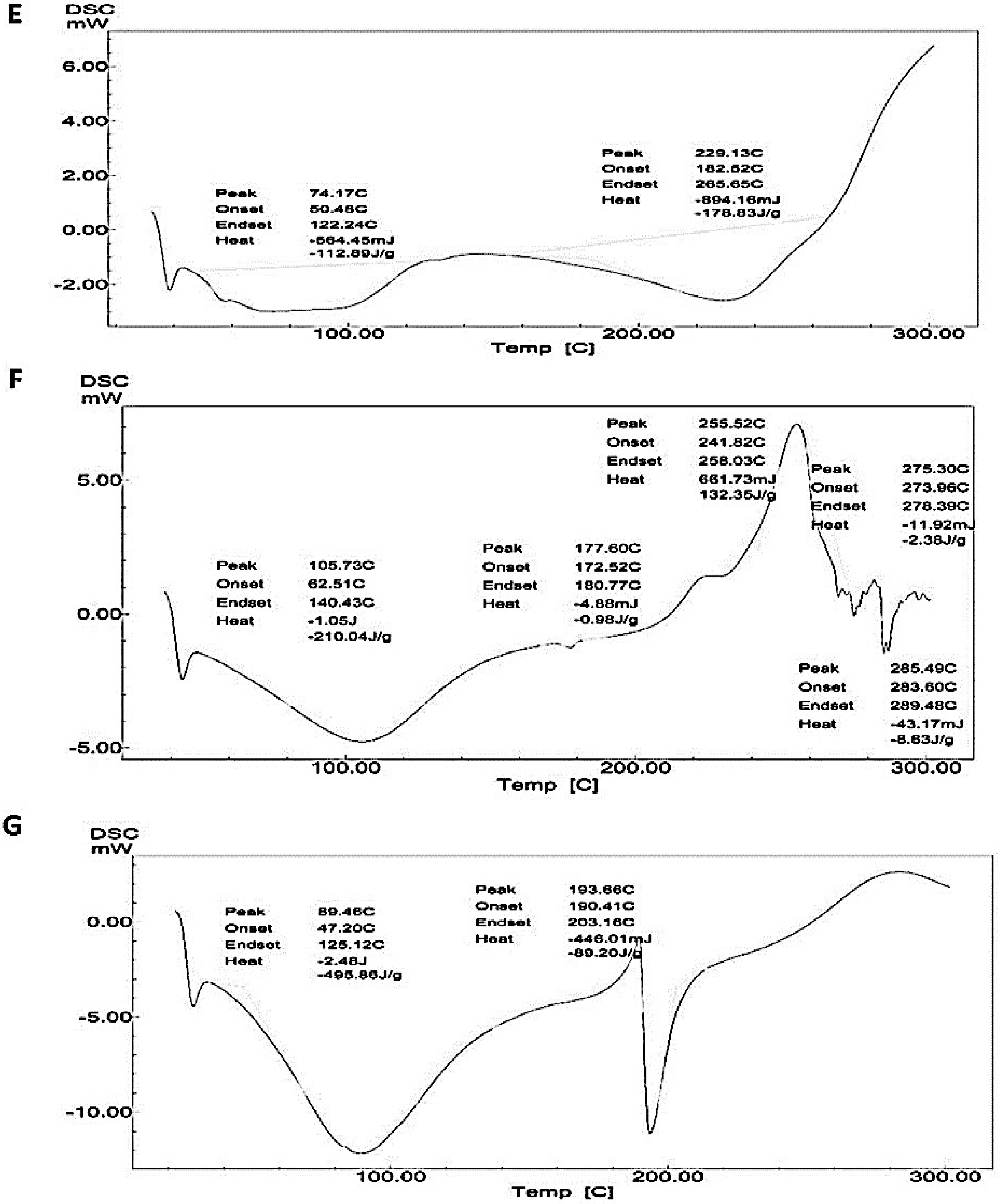

Figure S2**.** DSC spectra of the components of the complexes. A) MSZ-HP-β-CD inclusion complex, B) curcumin-HP-β-CD inclusion complex, C) Na-alginate, D) Chitosan, E) Eudragit® S-100, F) Optimized form a physical mixture and G) Optimized beads formula.

A) Data Set: placebo - RawData

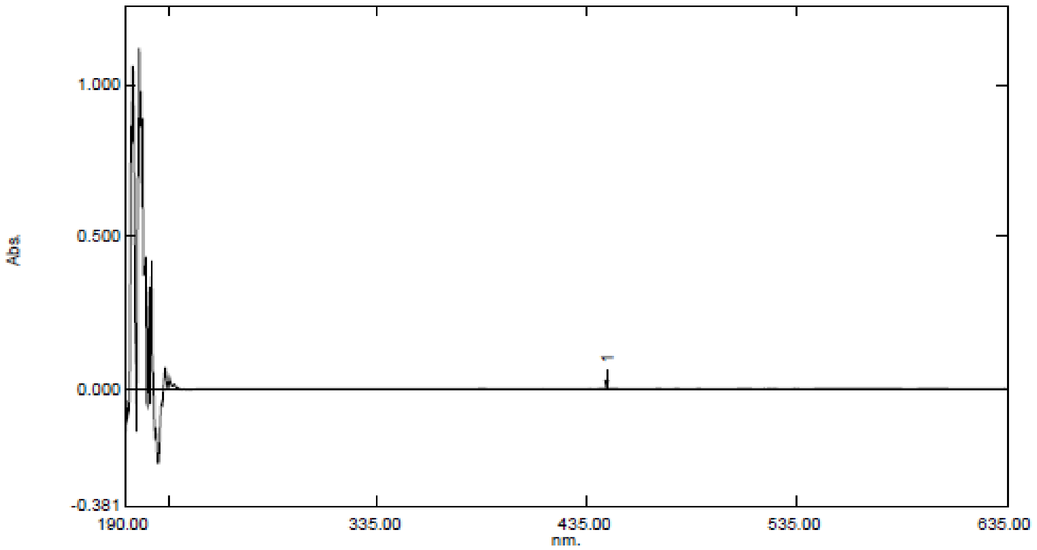

[Measurement Properties]
Wavelength Range (nm.): 190.00 to 635.00
Scan Speed: Fast
Sampling Interval: 0.2
Auto Sampling Interval: Enabled
Scan Mode: Auto

No.	Wavelength	Absorbance	Description
1	306.00	-0.000	
2	421.00	-0.000	

B) Data Set: System suitability 01 - RawData

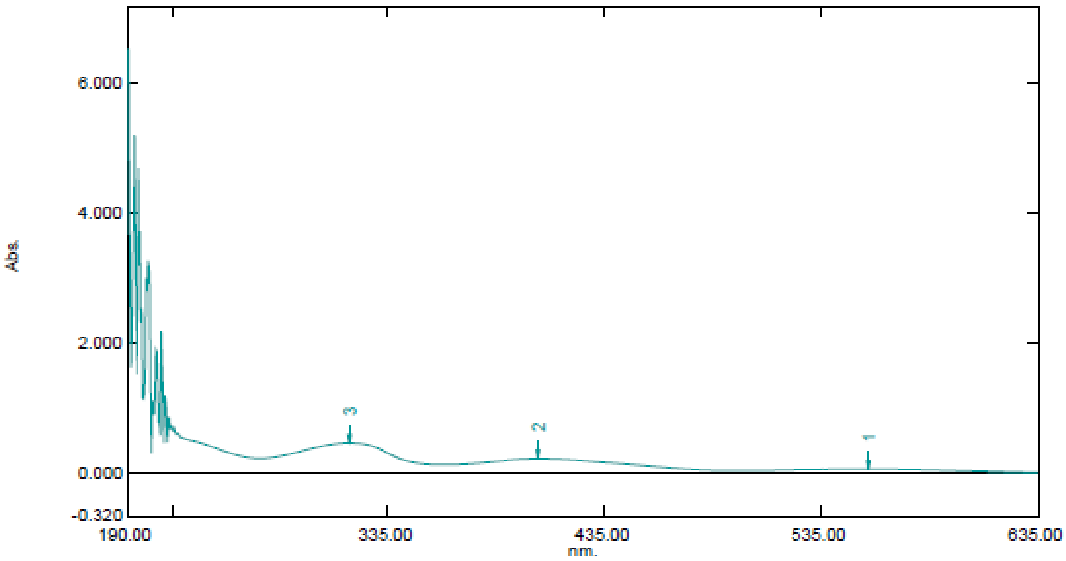

[Measurement Properties]
Wavelength Range (nm.): 190.00 to 635.00
Scan Speed: Fast
Sampling Interval: 0.2
Auto Sampling Interval: Enabled
Scan Mode: Auto

No.	Wavelength	Absorbance	Description
1	306.00	0.865	
2	421.00	0.423	

Figure S3. A) UV-Vis spectrum of beads plain formula with measurements at 306 and 421nm showing no absorbance. B) Standard mixture of MSZ and CUR for comparison.

A)

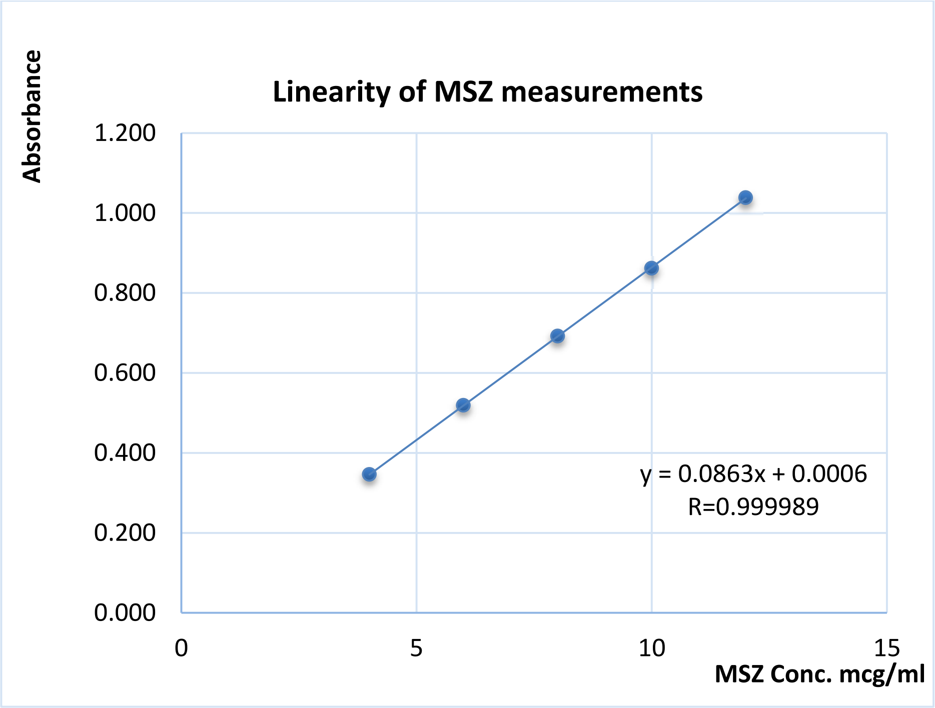

B)

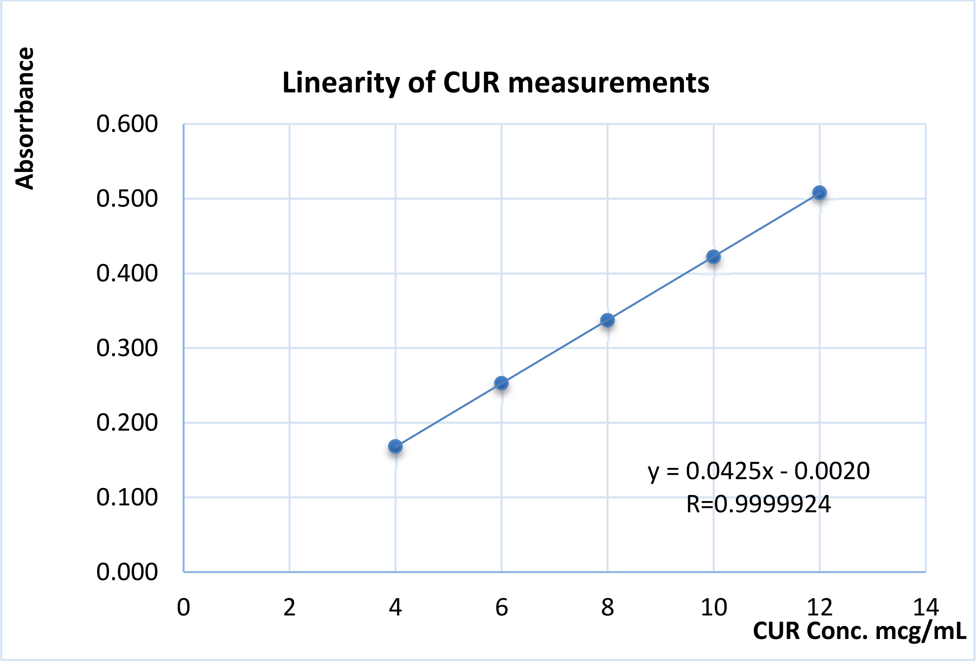

Figure S4. A) Linearity of MSZ UV-Vis Measurements at 306nm, B) Linearity of CUR UV-Vis Measurements at 421nm
